# Quantifying the Ocean, Freshwater and Human Effects on Year-to-Year Variability of One-Sea-Winter Atlantic Salmon Angled in Multiple Norwegian Rivers

**DOI:** 10.1371/journal.pone.0024005

**Published:** 2011-08-29

**Authors:** Jaime Otero, Arne J. Jensen, Jan Henning L'Abée-Lund, Nils Chr. Stenseth, Geir O. Storvik, Leif Asbjørn Vøllestad

**Affiliations:** 1 Department of Biology, Centre for Ecological and Evolutionary Synthesis (CEES), University of Oslo, Oslo, Norway; 2 Norwegian Institute for Nature Research (NINA), Trondheim, Norway; 3 Norwegian Water Resources and Energy Directorate (NVE), Oslo, Norway; 4 Institute of Marine Research (IMR), Flødevigen Marine Research Station, His, Norway; 5 Department of Mathematics, University of Oslo, Oslo, Norway; University of Hull, United Kingdom

## Abstract

Many Atlantic salmon, *Salmo salar*, populations are decreasing throughout the species' distributional range probably due to several factors acting in concert. A number of studies have documented the influence of freshwater and ocean conditions, climate variability and human impacts resulting from impoundment and aquaculture. However, most previous research has focused on analyzing single or only a few populations, and quantified isolated effects rather than handling multiple factors in conjunction. By using a multi-river mixed-effects model we estimated the effects of oceanic and river conditions, as well as human impacts, on year-to-year and between-river variability across 60 time series of recreational catch of one-sea-winter salmon (grilse) from Norwegian rivers over 29 years (1979–2007). Warm coastal temperatures at the time of smolt entrance into the sea and increased water discharge during upstream migration of mature fish were associated with higher rod catches of grilse. When hydropower stations were present in the course of the river systems the strength of the relationship with runoff was reduced. Catches of grilse in the river increased significantly following the reduction of the harvesting of this life-stage at sea. However, an average decreasing temporal trend was still detected and appeared to be stronger in the presence of salmon farms on the migration route of smolts in coastal/fjord areas. These results suggest that both ocean and freshwater conditions in conjunction with various human impacts contribute to shape interannual fluctuations and between-river variability of wild Atlantic salmon in Norwegian rivers. Current global change altering coastal temperature and water flow patterns might have implications for future grilse catches, moreover, positioning of aquaculture facilities as well as the implementation of hydropower schemes or other encroachments should be made with care when implementing management actions and searching for solutions to conserve this species.

## Introduction

Populations of animals exhibit fluctuations over a number of spatial and temporal scales. The underlying mechanisms of this variability comprise complex interactions between endogenous and exogenous processes for which relative importance varies among systems [1]. Variability is of particular significance for exploited species that can show boom-and-bust cycles as a consequence of harvesting-derived effects that destabilize population dynamics by altering demographic parameters. In line with this, it has recently been demonstrated that fishing increases the temporal variability of exploited fish stocks subjected to overharvesting due to the truncation of the age/size structure [2]. Diverse life history and local adaptations may, however, play a role in providing long-term stability and sustainability, and large-scale natural variations in environmental conditions are likely buffered by maintaining such biocomplexity (e.g. Pacific salmon [3]). However, additional non-natural factors attributable to human impacts may test the resilience of fish stocks such as salmonids. Composite effects like these have seldom been addressed in large-scale studies of salmonids population abundance.

Wild Atlantic salmon (*Salmo salar*) populations have been decreasing throughout their geographic distribution raising concern due to the species' economic and conservation importance. A number of factors causing severe declines and even extinctions have been identified although discerning individual mechanisms is often difficult due to their likely action in concert [4]. Changes in stocks have been associated with a broad spectrum of environmental factors at most time scales [5] and analyses of multiple populations revealed the importance of local-scale effects [6]. Here, we focus on quantifying how one-sea-winter fish rod catches vary at the river level in relation to oceanic and freshwater conditions and human stressors using multiple time series. As for most fisheries time series, the data probably contain mixed information on environmental variability, population dynamics and exploitation. However, the catch data can be used to test different effects through statistical modeling and detect overall signals on the observed fluctuations that are not necessarily dependent on the intrinsic properties of effort-corrected time series.

The life history of Atlantic salmon in Norwegian rivers is complex. Spawning occurs in freshwater around October–January. Juvenile life stages live in freshwater for 1–8 years before smolting and leaving their rivers to pursue oceanic feeding migrations. Post-smolts spend 1–4 years at sea before attaining sexual maturity, and returning (May–October) with high precision to their natal areas to spawn [7]. Individuals that become sexually mature after a single sea winter (1SW) are termed as grilse. Numerous factors potentially influence the different life history stages. In freshwater, density-dependence and predator-prey interactions play a fundamental role in shaping populations [8], and spatial habitat structure affects population dynamics and carrying capacity [9]. Later on, in the marine environment, it is believed that the largest component of natural mortality occurs during the first year at sea and climatic conditions will affect this phase in several ways [5]. Post-smolt survival has often been associated with sea surface temperature, which presumably modulates growth rates and controls recruitment [10]. Marine predators may also contribute to shaping population variability at the smolt stage [11] and/or on the return phase [12].

In addition to environmental conditions, salmonids must face several human-caused obstacles and stressful factors during their life cycles. Damming of the rivers alters entire ecosystems and declines in productivity, survival or growth have been ascribed to this threat [13]. However, recent studies show that smolt survival during their migration to sea is not lower despite the large presence of dams on the main stem of the rivers [14]. Differences in delayed mortality at sea seem to be, as well, non-detectable when comparing in-river and early ocean survival between populations with different smolt to adult return rates and migrating past a different number of dams [15]. Other human impacts include both coastal and oceanic fisheries causing, for instance, well-known structural changes in the spawning run of Norwegian populations observed after the ban of the drift net fishery in 1989 [16]. Furthermore, the exponential increase of salmon aquaculture with open net pens located in coastal waters might have contributed to the general decline of wild populations. Negative impacts associated with farmed salmon are well established and include, for instance, the reduction of fitness in wild salmon due to interactions with escaped individuals [17], and the increased mortality of wild populations due to parasite transmission (see review by [18]).

Nevertheless, despite the accumulated understanding of impacts on Atlantic salmon abundance, previous research has focused on single or small sets of rivers, and also tending to examine the effects of single variables over a specific life stage rather than the composite of effects that combine to determine population abundance towards the end of the life cycle. Therefore, the overall objective of this study is to quantify the oceanic and freshwater effects, as well as the human impacts on Atlantic salmon. We do this by analyzing time series of grilse rod catch throughout the range of Norwegian rivers. Based on the prior knowledge of the multiple factors that affect the survival of Atlantic salmon we used a multi-river mixed-effects model to investigate the following questions: What appears to be the main factors driving changes in grilse catches considering multiple populations at once? Are there differences across populations in the effects of oceanic and freshwater conditions? Is there evidence for cumulative impacts, especially human-caused impacts? To address these issues we run our statistical models across a unique dataset of 60 time series spanning from 1979 to 2007 of salmon caught in the rivers after a single winter at sea.

## Methods

### Catch data

**Figure 1:**
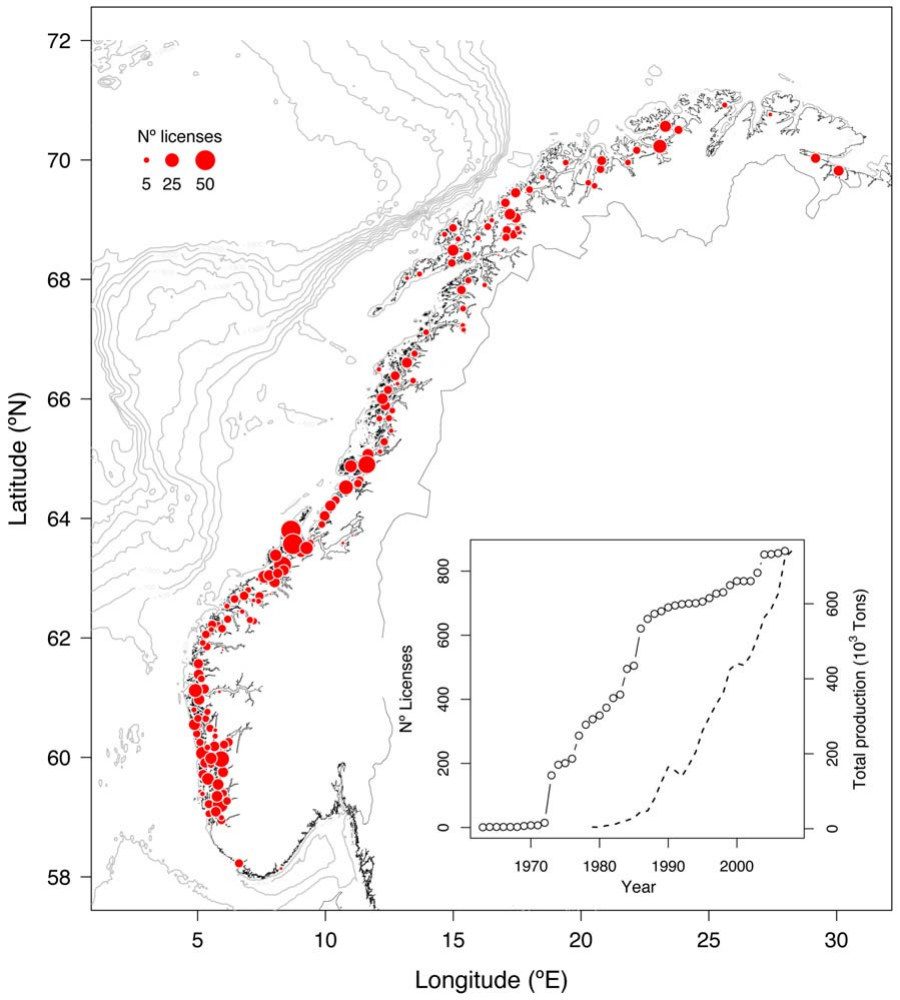
Map of the study area. It shows the distribution of salmon farming along the Norwegian coast, where the size of the dots indicates the cumulative number of licenses (i.e. companies) registered to operate in each municipality up to 2007 (data from the Directorate of Fisheries). Note that a given license might operate in different municipalities. Note also that each dot is positioned by averaging the coordinates of all net pens installed in each municipality by each license. The inset shows the cumulative time trends of farming licenses (open dots) according to the registered year of starting activities (data from the Directorate of Fisheries), and the total farmed salmon production (broken line) in Norway (data from SSB).

The present study is based on the official statistics of nominal catch of adult Atlantic salmon for the period 1979 to 2007 over a wide geographical range of Norwegian rivers (58°19′–70°37′ °N and 5°07′–30°32′ °E; Fig. 1). In Norway, the landowners own fishing rights and they, or their organizations, define the fishing rules for their areas. In addition there are national and regional rules implemented by the Norwegian authorities (The Directorate for Nature Management, DN, http://www.dirnat.no/; and County Governors). The legal fishing season is restricted to summer and early autumn, but differs somewhat among rivers. The fishing is performed by recreational anglers using rod and different kinds of tackles (e.g. flies, baited hooks or various kinds of lures). In general, one hook per rod is allowed. In the period studied here fishing with fixed gears (i.e. nets and traps) and seines have been banned in the majority of Norwegian rivers. The exception to this is three of the rivers used here (Tanaelva, Neidenelva and Numedalslågen) (see further details in [19]).

All individuals fishing for anadromous salmonids have to pay a national license. During 1996 to 2010 around 80 600 (±7600 SD) people in Norway paid the license for angling salmonids in freshwater with a slight decrease (∼1.4% yr^–1^) in numbers (DN, pers. comm.). However, to our knowledge, there is no detailed effort data for each Norwegian river considered in this study. Moreover, available information does not show broad changes in fishing pressure. For instance, in the river Altaelva, the number of daily licenses has remained quite stable (c.v. ∼13.2%) for most of the watercourse during the period 1982–2006 [20]. In addition, catches per unit of effort (n° salmon caught per hour or per daily license) are highly correlated (*r* ^2^>0.63) with salmon catches in this river (Ugedal O., pers. comm.). The most complete information is for the Finnish part of the river Tanaelva (note that this river is on the border between Norway and Finland, but catches from both parts are highly correlated, *r* ^2^∼0.7, and fishing days are correlated as well) from 1979 to 2007 [21], [22]. During this period the number of recreational fishermen varied approximately between 4000 and 10 000 (with a slight increase of 1.6% yr^–1^), and the number of fishing days varied between 12 000 and 38 000. Furthermore, similar to river Altaelva, there is a strong positive relationship (*r* ^2^∼0.8) between catches and catches per unit of effort (fishing effort measured as number of recreational fishermen or fishing days).

In Norway, systematic collection of data on salmonid fisheries (Atlantic salmon, sea trout *Salmo trutta*, and sea char *Salvelinus alpinus*) began in 1876 resulting in a database with annual catch data for more than 500 rivers. The fishermen report the catches to the landowners who then are responsible to send the reports to the county authorities that, in turn, compile the data for each watercourse and forward the results to the Norwegian official statistics agency (Statistics Norway, Statistisk Sentralbyrå, SSB, http://www.ssb.no/). However, landowners have not reported any effort information as discussed above. Starting in 1979, Atlantic salmon were identified at the species level and the total number and weight of different weight categories (<3 kg and ≥3 kg) has to be reported. The smaller weight group (<3 kg) mainly corresponds to one-sea-winter (1SW) fish (grilse), and the larger group corresponds to multi-sea-winter (MSW) fish (2SW, 3SW fish etc) [16]. Some bias may be introduced by using this classification, but we believe this is of minor importance as has been discussed elsewhere [23]. In this study we focused on grilse catches, and the analyses were based on the kilograms of grilse in the reported annual catch within each river. The rivers in the database varied considerably in size, as did the catches. In some rivers, catch was very low in some years, either because of small catches or because of problems with the reporting procedure. Therefore, we considered only rivers with complete continuous records for ≥15 years of data. A minimum catch of 20 individuals per year was set for inclusion in the analyses. This filtering excluded very small rivers with low catches and/or incomplete and less reliable data resulting in a final set of 60 rivers for analysis (Table S1; Fig. S1). Missing values in some catch time series (Table S1) were interpolated using a procedure based on a singular spectrum analysis [24].

### Environmental data

Our prime interest was to relate grilse catches with potential factors shaping variability once the smolts have left the rivers. Therefore, covariates were selected as such. To test for effects of sea surface temperature (SST) experienced by smolts on the subsequent interannual variability in grilse catch, we computed temporal averages of SST during the time of smolt entry to the sea. We selected coastal grid cells (1°×1°) from the Comprehensive Ocean–Atmosphere Data Set (COADS, http://icoads.noaa.gov/) whose centers were located nearby the ocean entry point of a given river. SST was monthly averaged according to population-specific timing of smolt descent depending on latitude (Table S2), that is, May for rivers south of 63 °N; May–June for rivers between 63 °N–69 °N; and June–July for rivers north of 69 °N.

To test for effects of freshwater runoff we estimated variation for each river catchment. Water flow affects early life stages of salmon [25], and the upstream migration patterns and success are influenced by runoff (see review by [26]). Disentangling the effect of water discharge during specific periods in the life cycle is difficult due to strong collinearity in water flow among the relevant seasons and at various (year) lags (runoff during upstream migration is related with runoff during spawning [October–January], *r* ^2^∼0.8; and with water flow during early life [May–August], *r* ^2^∼0.9). Moreover, the length of the parr period and in turn smolt age varies between 1–6 years depending on river. Smolting rate and environmental effects during the early freshwater phase might affect the later grilse catch, but, on the one hand, identifying appropriate time lags on freshwater effects is rather complex as information on smolt age composition is not available for each river. On the other hand, smolts and/or egg counts were not available either for each river. Therefore, we considered average runoff in each river for the summer upstream migration months (June–August) coincident with the Norwegian recreational fishery season. In doing so, daily discharge (m^3^ s^–1^) for each river catchment was estimated using a spatially distributed version of the Hydrologiska Byråns Vattenbalansavdelning model (HBV, http://www.smhi.se/foretag/m/hbv_demo/html/welcome.html) developed by the water balance section of the Swedish Meteorological and Hydrological Institute (see [27] and references therein). The model performs water balance calculations for 1-km^2^ grid cell elements that are characterized by their altitude and land use. It was run with daily time steps, and data inputs were precipitation and air temperature. Daily discharge data for the individual grid cells were subsequently aggregated to monthly runoff for the respective catchments. The model was calibrated with available information about climate and hydrological processes from gauged catchments in different parts of Norway, and parameter values were transferred to other catchments based on a classification of landscape characteristics. The model presents the ‘best’ simulations of runoff in each catchment without taking into account any specific hydrological regulation, damming or other kinds of alterations.

### Human impacts

Several human impacts could affect Atlantic salmon during the ocean life and subsequent river ascent. To determine the potential impact of salmon farming in net pens on smolts on their way to the open ocean, we compiled data on the presence of registered salmon farming companies (licenses) in each Norwegian municipality from the Directorate of Fisheries (http://www.fiskeridir.no/fiskeridir/akvakultur/registre, accessed on 22/June/2009) (Fig. 1). Each company runs multiple net pens that might be located in different municipalities and not containing fish simultaneously. Data on production at local scales is only available to the regional and national controlling authorities, and is not reported except as aggregated values on larger scales. Ideally, the data should reflect the total annual production of farmed salmon –and/or parasite abundance– per net pen but, unfortunately, to our knowledge, these data are not available. Therefore, due to these data limitations and to be conservative we used presence/absence of registered licenses taking into account the fact that aquaculture operations were established at different times across municipalities, and that a given license can have net pens in different (neighboring) municipalities, i.e. a license operating in two municipalities is assumed to affect rivers draining in both municipalities. Twenty-nine of 60 rivers were found to drain into coastal areas where at least one license was assumed to be active during any year of the study period.

Atlantic salmon has been exploited in the sea for many years using different types of nets. In Norwegian home waters several restrictions and management measures have been introduced but in general their effects have not been evaluated by follow-up studies (but see [16]). To examine the relationship of the coastal net fishery with the river catches we estimated the proportion of grilse caught at sea relative to the total kg of grilse caught at sea and in the rivers landed in each Norwegian county each year. The data were obtained from the Norwegian official statistics agency (SSB) that is in charge of compiling and verifying the catch reports sent by the sea fishermen by the end of the fishing season.

Finally, to estimate how the presence of hydroelectric dams might impact upstream migration and/or catch, we compiled data from the Norwegian Water Resources and Energy Directorate (http://www.nve.no/) on the presence of hydropower stations in each river. Hydropower development may differ strongly among schemes, for instance, in terms of water diverted and stored, positioning, size, management, etc. Therefore, to use a simple metric of hydroelectric effects we considered the presence of all dams that potentially affect water runoff within the main course of the rivers. Thus, 28 out of 60 rivers contained at least one hydroelectric scheme along the salmon-producing part of the river.

A summary of all the input variables is provided in Table S3.

### Statistical analyses

Data were analyzed using a restricted maximum likelihood (REML) linear mixed-effects model (random grouping factor comprises 60 rivers with 1707 observations) following methods described in [28]. This approach is appropriate for modeling multiple time series simultaneously and assumes that the rivers examined represent the population of all rivers. Model selection for selection of explanatory variables, random effects and correlation structures on error terms were all performed using the Bayesian Information Criterion (BIC) that penalizes more a model with higher number of parameters. Main effects were always included if interaction terms involving these effects were selected.

A preliminary analysis consisted of fitting separate linear models per river to choose parameters to account for between-river variation. Displaying confidence intervals on intercepts and slopes suggested that a random effect might be required to account for river-to-river variability in the intercept, and time and runoff slopes. Model selection analysis supported including random effects on the intercept and time (Year) slope (Table S4). According to these preliminary results we fitted a model of the form:
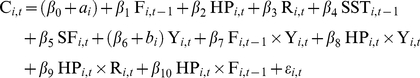
(1)


where C is the natural log-transformed catch of grilse for each river *i* at a time *t*. *β*_n_ are the fixed effects with covariates as follows: F is the absence (0) or presence (1) of fish farms (dichotomous variable), HP is the absence (0) or presence (1) of hydropower stations (dichotomous variable), R is the natural log-transformed runoff (continuous variable), SST is sea surface temperature (continuous variable) with a one year lag to accommodate effects on smolts during time of entry to the sea, SF is the sea fishery (continuous variable), and Y is year (continuous variable). *a* and *b* are the random river (*i*) effects for the intercept and time slope assumed to be independent for different rivers and to follow a normal distribution with mean zero and variances 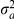 and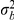, respectively; and 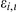 is the observation error assumed to be independent and normally distributed. The presence of farms and hydropower stations could have cumulative impacts on the catches that would be reflected on their interactions with the time trend. Both effects could, as well, interact with each other. Furthermore, the water flow is expected to vary with the presence of dams. Covariates were centered by subtracting the mean before running the models. Population time series are often autocorrelated as a result of the autocorrelation in the environmental variables and of demographic effects, thus the independence assumption on the error may be incorrect. Different autoregressive moving average (ARMA) structures were tested to model within-group serial correlation in 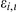 (Table S4). BIC indicated that an ARMA model of order 1 [ARMA(1,1)] provided the better fit of the data (Table S4), i.e:
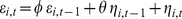
(2)where 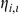 is independently normally distributed noise: 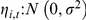


Finally, variance in residual catches (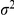) was modeled as an exponential function of runoff (R) that allows for an observed decrease of spread of this covariate, i.e.:
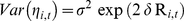
(3)


where 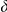 is an unknown parameter to be estimated that describes the estimated change in variance with runoff (R).

The model we applied assumes that there is no correlation of residuals for the different catch time series. However, this assumption could be violated if, for instance, there is spatial correlation in other environmental variables not included in our model. In order to assess this assumption we computed the Spearman's correlation coefficient for each pair of rivers' normalized residuals (i.e. calculating the correlation coefficients between the 60 residual time series which leads to 1770 possible combinations) and evaluated if the remaining temporal fluctuation of rivers were structured in space. In doing so we used the Mantel test to investigate whether the similarity (our metric of similarities was the Spearman's correlation coefficient) among the 60 residual time series were independent of the geographical location of the rivers, and the Mantel correlogram to assess the spatial scaling [29]. In addition to the matrices comparison, we evaluated the individual pairs of correlations in more detail. Performing correlations on each pair of residuals time series induces multiple testing, i.e. the probability of making Type I errors is larger than the significance level (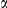). Therefore, to reduce the probability of deciding that a correlation is significant when it is random we applied Bonferroni correction for adjusting significance levels.

All analyses were performed on R 2.6.2 language [30] and using the “nlme 3.1-86” package [28].

### Sensitivity analyses

Due to some particularities of the data set we performed sensitivity analyses. First, we tested the robustness of the final model to several exclusions of data (see Discussion S1 for a detailed description). Second, as mentioned above, data on fishing effort for each river was not available, though some rivers' information –and the last 15 y for the whole country– revealed non broad changes on fishing pressure during the studied period. Therefore, on the one hand, it could be plausible to assume that the low variability in effort would not affect the catches; on the other hand, given the model formulation, part of the variability in the catches that could be attributed to fishing effort –not included in Eq. 1– would end up in the random component of the final model. Despite these considerations we performed a simulation exercise to test the robustness of the coefficients of the final mixed model. First, we fitted an integrated ARMA (ARIMA) model [31] to the number of fishing days in the Finnish part of river Tanaelva for the period 1979–2007. Second, we assumed that fishing effort in each river follows the same structure as Tanaelva; therefore, we used the coefficients of the optimal ARIMA model –fitted to Tanaelva effort– to simulate time series of fishing effort for each of the 60 rivers (see Discussion S1). The simulated effort was then included as a new covariate in the final mixed model (i.e. *β*_11_ Effort*_i_*_,*t*_ in Eq. 1) without changing its formulation. In total, we simulated 60 000 different time series of effort, that is, we ran the model including effort as a new covariate 1000 times.

## Results

Catches were highly variable from year to year in all rivers (Fig. S1) with a mean value of 1776 kg ranging from 25 to 45 020 kg. Average SST was 8.09°C (5.83–13°C), and runoff ranged from 0.69 to 1618 m^3^ s^–1^ averaging 99.16 m^3^ s^–1^ (Table S3). In addition, farming licenses were widespread distributed along the Norwegian coast with large aggregations on the Central and Western regions (Fig. 1). No signs of collinearity between covariates were apparent (Variance Inflation Factors <1.69).

Model selection analysis indicated that the optimal model includes a random effect for the intercept and the time (i.e. Year) slope to account for between-river variability (Table S4), though variation in the slope (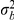) was relatively small. Table 1 shows the estimated parameters and hypothesis tests for the fixed-effects terms related to the optimal model. Coastal SST at the time of smolt entrance into the sea was positively related with the following year's catches of grilse in the river, implying an increase in catches by ∼4.6% 0.5°C^–1^. In addition, a 1% increase of estimated runoff during upstream migration would yield a ∼0.4% increase in the average catches although the significant interaction of water flow with the presence of hydropower stations reflects a lower average trend (i.e. ∼0.11%) when hydropower stations are present (Table 1). Furthermore, we also found evidence for changes in catch variation related to runoff as supported from the variance model. The estimated exponential variance parameter (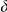, Table 1) indicates that grilse catches were narrower with increased runoff during upstream migration corresponding to a 13.3% decrease in variance with a 2 ln(m^3^ s^–1^) increase in runoff. Moreover, river catches of grilse increased significantly when harvesting of this life-stage at sea was reduced. This implies that a 1% decrease of 1SW catches at sea leads to a later increase of ∼1.7% of 1SW catches at rivers.

**Table 1:** Optimal model results.

Effects	Estimate	95% CI	*t*-value	*P*-value
Fixed				
Intercept	6.607	6.271; 6.943	38.568	<0.0001
F	0.349	0.017; 0.681	2.064	0.0392
HP	–0.105	–0.496; 0.285	–0.529	0.5966
R	0.406	0.283; 0.529	6.469	<0.0001
SST	0.092	0.055; 0.129	4.911	<0.0001
SF	–0.017	–0.020; –0.014	–10.242	<0.0001
Y	–0.011	–0.025; 0.002	–1.642	0.1008
F×Y	–0.035	–0.054; –0.017	–3.812	0.0001
HP × R	–0.299	–0.471; –0.127	–3.416	0.0007
Random (SD)				
Intercept ( 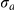 )	0.898	0.738; 1.091	na	na
Y ( 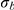 )	0.022	0.013; 0.039	na	na
Residual ( 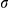 )	0.737	0.680; 0.800	na	na
Correlation structure				
align="left" valign="top"> 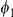	0.717	0.581; 0.814	na	na
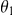	–0.299	–0.412; –0.177	na	na
Variance function				
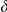	–0.036	–0.057; –0.014	na	na

CI: confidence interval; SD: standard deviation; na: not applicable.

Parameter estimates and statistical significance from the optimal mixed-effects model with River as random grouping factor (60 levels). Abbreviations and units are described in the text and Table S3. Note that when dichotomous variables are involved the baseline case for comparison is the absence of farms and hydropower stations.

**Figure 2:**
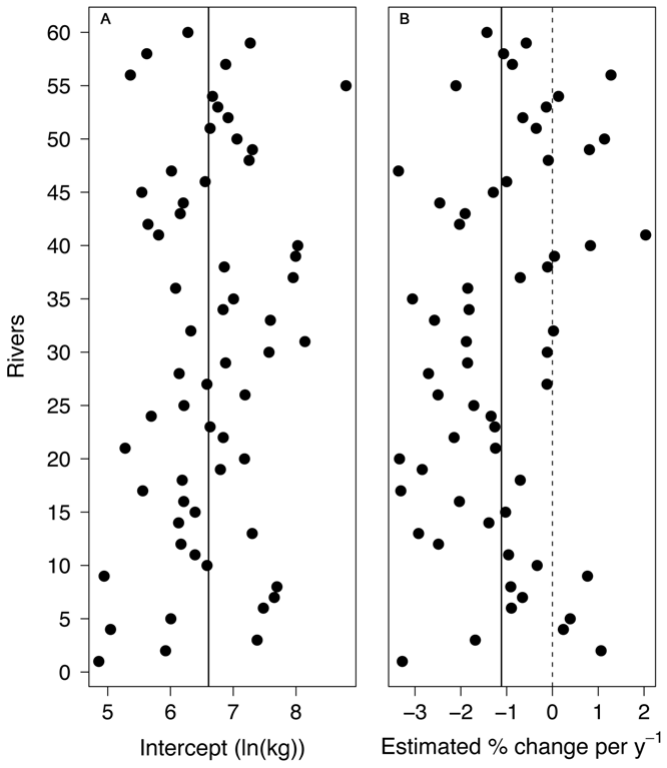
Estimated random effects. (A) River-specific intercepts (vertical solid line shows the average Intercept, *β*_0_ in Eq. 1; and dots show the river-specific intercept, *β*_0_+*a_i_* in Eq. 1) representing the predicted catch given the mean value of all covariates and absence of farming and hydropower development. (B) Estimated percentage change in catches of grilse per year (vertical solid line shows the average Year effect, *β*_6_ in Eq. 1; and dots show the river-specific random effects, *β*_6_+*b_i_* in Eq. 1). Rivers along the *y*-axis are ordered from south to north with the numbers corresponding to those in ‘ID’ column in Table S1.

**Figure 3:**
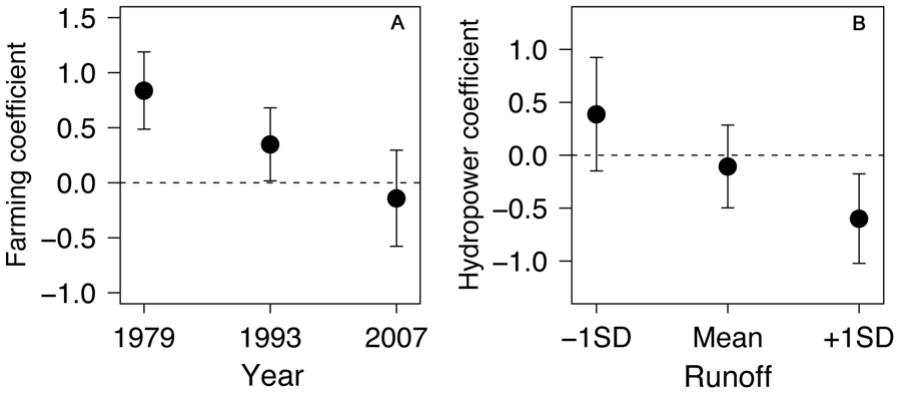
Main effects of farming and hydropower with different centering. (A) Main effect of farming (*β*_1_ in Eq. 1) obtained from models with different year centering (subtracting the min, mean and max). (B) Main effect of hydropower development (*β*_2_ in Eq. 1) obtained from models with different runoff centering (subtracting the mean and ±1SD). The bars represent 95% confidence intervals.

Estimated random intercepts do not suggest a strong spatial structure in average catches, although Tanaelva river is highlighted as the most productive one (Fig. 2A). In general, an average decreasing temporal trend in catches (∼1.1% yr^–1^, Table 1) was detected but with some rivers in the northern and southern extremities of the range of distribution showing increasing patterns (Fig. 2B). The interaction of Year with the presence of farms was also significant, reflecting a steeper average decrease (∼4.6% yr^–1^) with the presence of salmon farms in the corresponding draining areas (Table 1). However, the presence of hydropower stations did not interact with the time trend (*β*_8_ in Eq. 1 was not significant, thus removed from the model when selecting the optimal fixed structure). To further investigate the interactions we reran the model with different centering and found that catch in rivers with presence of salmon farms compared to rivers without (*β*_1_ in Eq. 1) was significantly higher during most of the study period, but the difference in catches between rivers draining in areas with presence of aquaculture operations and absence of salmon farms diminished with time (Fig. 3A). Furthermore, at low values of runoff catches were higher when dams were present compared to the absence of hydropower stations (*β*_2_ in Eq. 1). However, at high values of runoff the catch was lower with the presence of dams (Fig. 3B). Finally, we did not find strong support for an interaction between the presence of farms and hydropower stations (*β*_10_ in Eq. 1 was not significant and was therefore removed from the model when selecting the optimal fixed structure).

**Figure 4:**
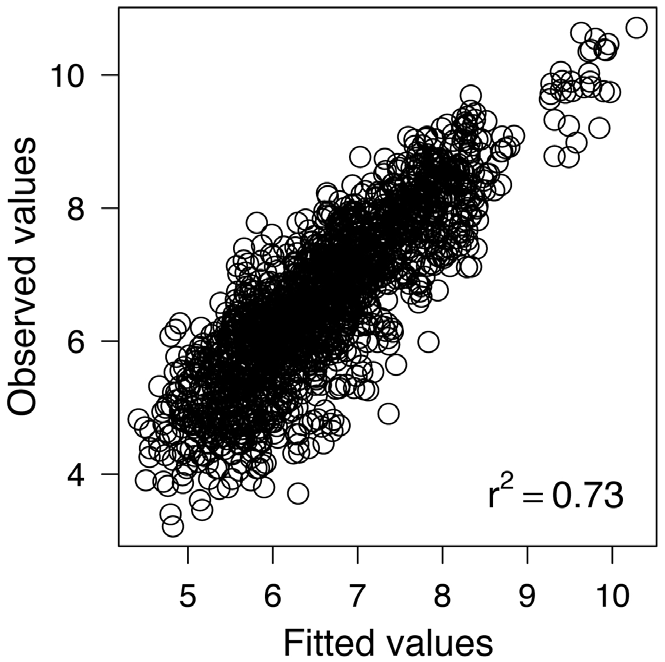
Model evaluation. Observed versus within-group fitted values plot for model depicted in Table 1. Note that the values on the extreme upper right side of the figure correspond to Tanaelva, which is the largest river along Norway in terms of catches (see Fig. 2A, and Table S1).

The positive and negative coefficients of the AR and MA components (Table 1) indicate strong positive autocorrelation at lag one. Figure 4 shows the observed values versus the predicted catches from the model depicted in Table 1. Within-group residuals were normally distributed and do not show any apparent variability. Random effects were also normally distributed and independent (Fig. S2). Furthermore, there were neither strong evidences of heterogeneity of residuals by river (Fig. S3), nor remaining temporal correlation (Fig. S4). Post-hoc analysis of spatial dependencies revealed that the similarities among the residual time series slightly depend on the geographical distance (Mantel *r* = 0.26, *P*<0.001; the level of significance was evaluated performing 10 000 permutations) with the highest similarities occurring at the smallest scale (Fig. S5). However, the Spearman's correlation coefficients for each pair of rivers' residual time series were strongly non-significant after Bonferroni correction (only 8 out of 1770 correlations were significant, Fig. S6). Therefore, the assumed independence between residuals of the different time series seems to be appropriate, although, very few neighboring rivers are closely related.

**Figure 5:**
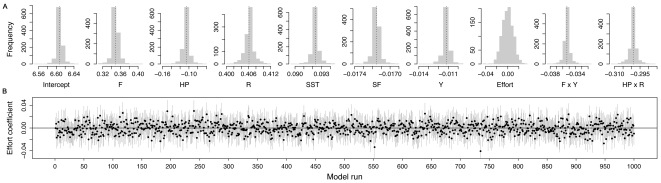
Simulation results. (A) Histograms of estimates of the coefficients with simulated effort included as a new covariate from the model runs as described in ‘Sensitivity analyses’ subsection. The vertical dotted lines indicate the estimates obtained using the model that does not include simulated effort (see specific values in Table 1). Abbreviations are described in the main text. (B) Value of effort coefficient and 95% CI in each model run. This coefficient is statistically significant if the CI does not include zero.

The sensitivity analyses did not change the results. On the one hand, the final model was robust to several exclusions of data (see Discussion S1 for a detailed description). On the other hand, simulated effort seemed not to play any relevant role. The most optimal time series model for fishing effort in river Tanaelva follows an ARIMA(3,1,0) process. The estimated model was 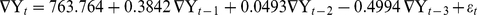 (note that 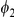 was not statistically significant) which standardized residuals were normally distributed and didn't show any temporal dependence. Using the above coefficients we simulated 1000 different time series for each river and ran the mixed-model 1000 times including simulated effort as a new covariate. Histograms of each coefficient showed very narrow variability and there were no major departures from the values obtained with the model that did not include any effort term (Fig. 5A). Moreover, ‘fishing effort’ coefficient was close to zero (Fig. 5A) and statistically non-significant. In 95.4% of the cases, out of 1000 model runs, ‘fishing effort’ was not significant (Fig. 5B).

## Discussion

Our analyses are focused on the effects of environmental factors and human impacts arising from the smolt stage onwards. Previous research has focused on analyzing data on catch or population numbers in single or only a few rivers, and seldom considering multiple factors in conjunction. Here, we used long salmon catch time series systematically gathered with a high geographical and temporal scope to explore signals in salmon variation and its potential causes. In the absence of direct experimental and/or observational work on mortality effects on salmon, and direct measures of abundance along the whole Norwegian range of distribution we consider that, despite some of the deficiencies described above (see subsection ‘Catch data’ in Methods, and Discussion S1), the catch data give a fairly correct picture of the yearly variation of Norwegian Atlantic salmon and represent a legitimate approach to make valid inferences. In this sense, we show that both oceanic and freshwater conditions are important for shaping the year-to-year variation of grilse catches in Norwegian rivers. Furthermore, the presence of aquaculture operations appears to strengthen the observed average decreasing trend in catches. In addition, the presence of hydropower stations in the rivers weakens the relationship between catches with the water flow during upstream migration. Taken together, the overall results are consistent and agree well with other studies using survival, or other metrics for abundance.

It has been shown that the production of returning adults is related to the number of descending smolts from which these adults were produced suggesting that density-independent factors are important for the ocean survival [32]. Marine conditions related to SST have been observed to affect post-smolt survivorship on both sides of the Atlantic, such that warmer SST in spring increases survival [33]. Increased survival also appears to be strongly growth mediated [34], and may be causatively linked [35]. Other studies have found negative relationships between midwinter (January) SST and growth condition during the subsequent summer return migration to freshwater [36] suggesting a prey-mediated mechanism coincident with the current decline in recruitment [10]. Recent research over wider spatial and time scales did not, however, find consistent correlations between salmon growth and environmental variability [37]. Anyhow, our results show a positive relationship between the amount of grilse caught in the rivers and the mean coastal SST at the time when smolts migrate to the sea (i.e. spring–summer one year before). Furthermore, this relationship is consistent throughout the latitudinal range examined (a random effect on SST was not supported) and the sign of the effect agrees with previous results across several Norwegian rivers [38], [39], and other rivers elsewhere (e.g. Pacific salmon in Alaska, [40]); but differs from what was shown in other areas (e.g. Pacific salmon in Washington and British Columbia, [40]; Atlantic salmon in the Northwest Atlantic, [41]). The strict timing of smolt descent would ensure precise alignment with the ‘optimal’ thermal habitat around 8°–10°C (note that 50% of our SST values range from 7.46 to 8.53°C). Moreover, recent results from Norwegian rivers suggest that the positive relationship between salmon abundance and seawater temperature would be indirectly linked through the match between the timing of the early marine phase with the plankton spring bloom [39]. In other areas, survival rates are positively and negatively related to SST fluctuations depending on latitude, suggesting underlying mechanisms that are related to the abundance of predators and prey likely associated with variations in coastal temperature that differs between oceanographic domains [40]. Therefore, oceanic conditions during early life at sea appear to be important for shaping interannual variability in survival and thus the number of returning grilse the year after. However, the contribution of (a) specific mechanism(s) during early sea life and its connection(s) with the effects in the subsequent months of open ocean living remains to be understood.

Upstream migration patterns in Atlantic salmon are complex and likely controlled by several factors. Water flow is reported as an important variable that stimulates and governs the spawning migration, though frequently constrained by other factors [26]. Our results show that increased water runoff during upstream migratory months is positively related with the amount of grilse caught in the rivers. However, the average slope of this relationship weakens by ∼74% when a hydropower station is present. Several studies have examined the effect of water discharge showing, for instance, positive associations between flow and swimming activity [42], and a body-sized dependence on runoff according to the size of the stream [43]. Upstream migration is physiologically demanding, and, to some extent, river migration success is related to water temperature [26]. Indeed, warmer water temperatures are generally associated with lower water discharges as would be the case in Norwegian rivers (Fig. S7).

Fish migrating upstream are vulnerable to the presence of several man-made obstacles on the fish's way to the spawning grounds. In fact, damming has been claimed as one of the most severe threats preventing recovery of endangered salmon populations [44]. However, the impacts seem to be river-specific for both Atlantic [45] and Pacific species [14], [46]. In line with this, on the one hand, we did not find an average steeper time drop in catches for those dammed rivers as has been shown for single river analyses [47]. On the other hand, we have identified that there is an average effect of runoff favoring the increase of catches, but the flow regime loses importance with the presence of a hydropower station in the course of the rivers. Our data indicate that those rivers with hydropower stations in their courses are larger, have higher average runoff, longer coastal distance, and length, and are less steep [23, Fig. S8]. Therefore, the presence/absence of hydropower stations is, to some extent, related to river size and migration arduousness. Taking this information into account, it seems that water flow is probably more important for upstream migration in relatively small rivers compared to larger rivers where runoff should not be a limiting factor (see also [26]). Moreover, this interaction is complex and can be regime specific, i.e. when runoff is low, average catches are higher in those rivers with presence of dams (i.e. larger rivers) compared to rivers without hydropower stations (i.e. smaller rivers); however, at high values of runoff this relationship reverses (Fig. 3B) suggesting that, on average, higher catches occur in smaller rivers with elevated levels of water flow. This could reinforce the fact that habitat characteristics play a relevant role [23], and points to the importance of site-specific responses to the same environmental variables as has been shown elsewhere [48], [49]. Finally, angler behavior has been revealed to be important in freshwater fisheries [50]. Regarding our analysis, it could be possible that water flow could be correlated with fishermen dynamics as there are anecdotal indicators that fishermen leave rivers without hydropower stations in favor of rivers with hydropower stations in summers with little precipitation. However, we consider that this effect would be probably of minor importance given that regulated rivers were dammed before the first year of the time series.

Salmon farming in Norway experienced an exponential expansion over the past 40 years reaching ∼750 000 tons and >800 licenses (inset in Fig. 1). However, in spite of finding a higher decreasing trend in catches with the presence of farming licenses, we cannot distinguish any concrete negative agent through the present analyses. Numerous studies have reported direct and indirect effects of farmed salmon on wild populations (see review by [51]), and global analyses have shown that migrating smolts that pass by net pens have dramatically reduced survival rates [52] presumably associated with lice infestations [18] but see [53]. Furthermore, strong negative genetically based effects of inter-breeding are well documented [17]. Our results also show that catches were higher under the presence of farms compared to the absence of aquaculture operations during most of the study period, though the difference narrowed in time (Fig. 3A). Although it is postulated that escaped farmed salmon enter Norwegian rivers late in the season [6], the former result could suggest, to some extent, that salmon numbers were higher in rivers draining in areas with farms due to the escapees, but decreasing in time. Indeed, the average proportion of escaped salmon in rivers monitored close to the spawning season has decreased from ∼30% during the late 1980s to ∼10% in more recent years [51]. Alternatively, this effect could reflect somehow the fact that fish farms were intentionally placed in the more productive areas.

Our model shows that catches of grilse in the rivers increased significantly with the reduction of harvesting this life-stage at sea. However, this effect should be interpreted with caution because data were available at a broad geographical scale using the sea catches from the same county as a covariate for a particular river, thus any catch in the coastal fisheries assumed to affect a given river that is not landed in the county of that river would underestimate the true covariate value for sea fishery. Moreover, there have been implemented different management rules of the coastal fisheries along the Norwegian coast not taken into account here that need further specific research. Nevertheless, the sea fishery can influence catches in the rivers as has been previously shown, for instance, after the ban of the drift net fishery in 1989 the catches, proportion and mean weight of grilse increased in various Norwegian rivers [16].

Finally, the ARMA structure of the error term could result from the correlation over time in the environmental fluctuations, as revealed by the moving average term; and from the life history strategy of the Atlantic salmon, as revealed by the autoregressive term. For instance, in mid- to late summer the growth trajectories in a sibling salmon population diverge, forming a group of potential emigrants and a group of resident individuals that will remain in freshwater for at least one more year before metamorphosing into the migratory smolt stage. This life history flexibility seems to be genetically based but environmentally driven developing a bimodal distribution of the juvenile salmon population [54]. Therefore, the temporal correlation could be consistent with the alternative smolting strategies adopted by individual salmons originated from the same cohort. Otherwise, it would reflect, to some extent, that young stages from two different cohorts experienced similar conditions during part of their freshwater living. Besides, we found that most of the residual patterns of the different time series are uncorrelated, however, a few rivers across the smallest spatial scale are closely related (only 8 out of 1770 combinations) suggesting, for instance, that a covariate is missing for those time series. An environmental effect later in the adult stage during the open ocean feeding could be, among others, a candidate for such covariates that deserves further research. Alternatively, it could be the case that fish from nearby rivers return along similar migration routes that take the fish past sea fisheries that might be underestimated (as commented above) and may generate residual correlation between two rivers if fish from both rivers share similar migration routes. Nevertheless, removing from the analyses those rivers with significant correlations shown in Figure S6 did not change our results, and any other correlation was no longer significant after adjusting the probabilities.

We can conclude then that year-to-year variability of grilse catch in Norwegian rivers is influenced by both oceanic and freshwater factors. Specifically, one-sea winter fish catches were positively related to SST at the time of entering the sea, however, current ocean warming might have future implications through alterations of the food web. Overall a decreasing trend in catches is apparent, and the presence of salmon farms in the coastal areas close to the rivers increases this depletion. In addition, water discharge benefited upstream migration of one-sea winter fish, however, in rivers with hydropower stations (usually larger rivers) the water flow loses importance suggesting that runoff might be a limiting factor in small rivers. Therefore, positioning of aquaculture facilities should be made with care, as well should the implementation of hydropower schemes or other encroachments that might influence water flow. Thus, this knowledge should be taken into account when implementing management actions and searching for solutions for solving conflicts between sectors, to conserve this species under the current global change and subjected to the impacts of multiple human threats [19].

## Supporting Information

Figure S1 **Time series of Atlantic salmon grilse caught in the 60 rivers analyzed.** Numbers correspond to ‘ID’ column in Table S1. Red dots highlight interpolated values for those time series with missing data. Note also that *y*-axis values differ among plots.
(TIF)

Figure S2 **Model validation.** Normality of the within-group normalized residuals (A), and scatterplot of the normalized residuals versus the fitted values (B). In addition, no significant patterns were found when plotting the residuals versus each explanatory variable (not shown). Normal plot (C) and scatterplot (D) of estimated random effects. YearC indicates ‘Year Centered’.
(TIF)

Figure S3 **Normalized residuals versus fitted values by river.** Numbers correspond to ‘ID’ column in Table S1.
(TIF)

Figure S4 **Partial Autocorrelation Function of the normalized residuals for each river.** Numbers correspond to ‘ID’ column in Table S1.
(TIF)

Figure S5 **Spatial similarities.** Mantel correlogram computed for 12 geographical distance classes on the 60 normalized residual time series. Positive values of the Mantel correlation indicate that similarity within that class of distance is higher than average, whereas, it is negative when the similarity is lower. Filled dots indicate significant results (*P*<0.05) of the Mantel test for a given distance class.
(TIF)

Figure S6 **Pairwise Spearman correlations between river's normalized residuals and geographical distance (i.e. 1770 possible combinations).** Open black circles indicate non-significant correlations; blue dots show significant correlations (301), and red dots show the significant pair correlations after Bonferroni correction (8; the corresponding rivers' number is indicated in brackets). The red line shows the fitted curve from a nonlinear model using generalized least squares (gnls). The average Spearman correlation was 0.16 ranging from –0.52 to 0.85 with 50% of the values between 0.02 and 0.31. Note also, that fitting variograms to the normalized residuals of the optimal model per year did not show any spatial correlation.
(TIF)

Figure S7 **Water temperature and runoff.** Plot of water temperature (°C) against runoff (m^3^ s^–1^) during upstream migration months (June–August) measured in six Norwegian rivers (Tovdalselva, Audna, Vosso, Gaula, Jølstra and Vefsna) from 1992 to 2007. The fitted curve from a biexponential model is also shown.
(TIF)

Figure S8 **Rivers' characteristics.** Box plots showing the variation in: (A) natural log-transformed catchment areas, (B) natural log-transformed mean water flow during upstream migration, (C) coastal migration distance (distance from river mouth to the coastal shelf), and (D) natural log-transformed steepness (ratio between altitude and river length) for the Norwegian rivers analyzed in this study with no hydropower stations (no HP) and the presence of at least one hydroelectric scheme along the salmon-producing part of the river (HP). The number of rivers with available data used in each plot appears on top of each graph. The asterisks indicate that differences are statistically significant according to an analysis of variance.
(TIF)

Table S1 **List of the rivers which interannual grilse catches were analyzed in this study.** Latitude (N) and longitude (E) give the geographical position of each river mouth. Overall mean ±SD (kg), and number of observations (years), with the number of missing years between parentheses, are also shown. The last column indicates the first year (yy) in the time series with hydropower development (HP) and/or salmon farming (F). ‘no’ denotes non-presence of that activity for a given river during the studied period.
(PDF)

Table S2 **Median dates as well as earliest and latest dates of smolt run of Atlantic salmon in some Norwegian watersheds over several years.** The descent date is defined as the day each year when 50% of the smolts have passed the gauging station.
(PDF)

Table S3 **Summary of the variables used in this large-scale analysis (** ***n*** **= 60 one-sea-winter Atlantic salmon time series).**
(PDF)

Table S4 **Comparison of different models showing the number of parameters, Bayesian Information Criteria (BIC) and the difference in BIC values between each model and the model with the optimal random structure.** First, we selected the appropriate random effects (Models 1 to 5) where the fixed component contained all explanatory variables and reasonable interactions. Then, different correlation structures (Autoregressive: AR, Moving Average: MA) for modeling within-group serial correlation were compared (Models 6 to 8), and heteroscedasticity was handled by modeling the residual variance as an exponential function of runoff (Model 9).
(PDF)

Discussion S1 **Further details on catch data and model robustness.**
(PDF)
