# Supplementary figures and images for: Quantifying the Ocean, Freshwater and Human Effects on Year-to-Year Variability of One-Sea-Winter Atlantic Salmon Angled in Multiple Norwegian Rivers
Source: PLoS One. 2011 Aug 29;6(8):e24005. doi: 10.1371/journal.pone.0024005 (PMC3163678; doi:10.1371/journal.pone.0024005)

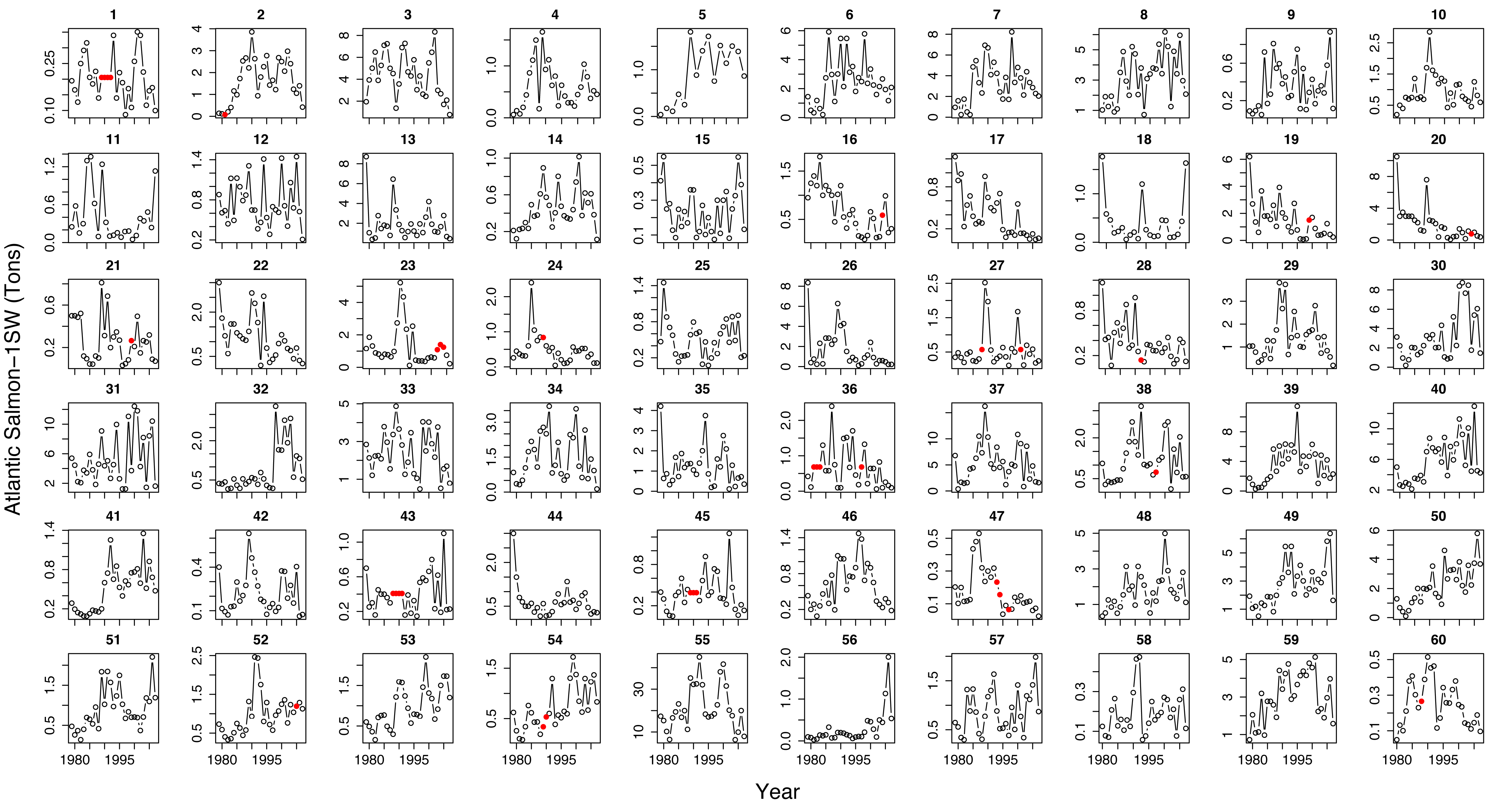

Supplement: Figure S1 — Time series of Atlantic salmon grilse caught in the 60 rivers analyzed. Numbers correspond to ‘ID’ column in Table S1. Red dots highlight interpolated values for those time series with missing data. Note also that y-axis values differ among plots. (TIF) [file pone.0024005.s001.tif]

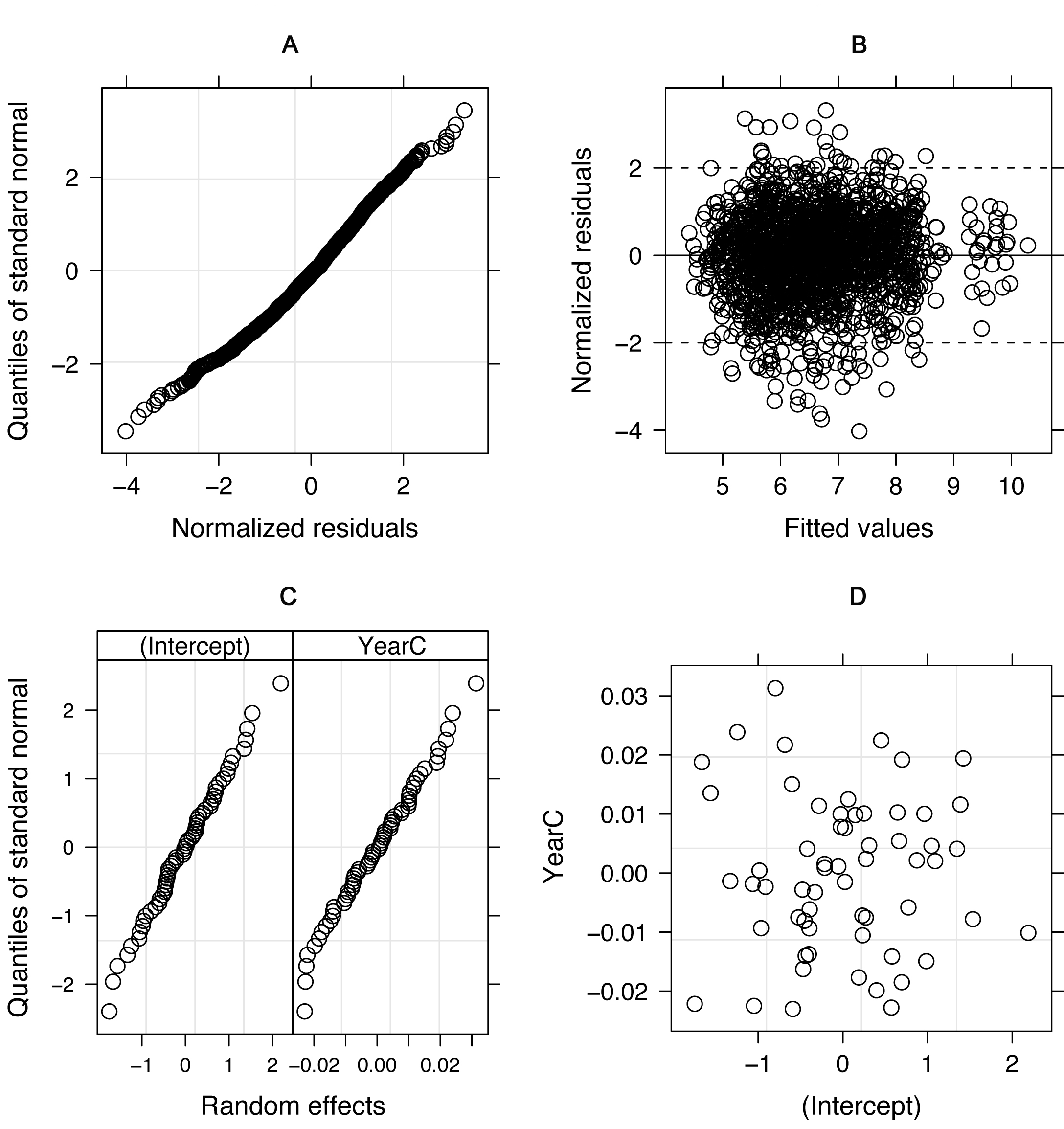

Supplement: Figure S2 — Model validation. Normality of the within-group normalized residuals (A), and scatterplot of the normalized residuals versus the fitted values (B). In addition, no significant patterns were found when plotting the residuals versus each explanatory variable (not shown). Normal plot (C) and scatterplot (D) of estimated random effects. YearC indicates ‘Year Centered’. (TIF) [file pone.0024005.s002.tif]

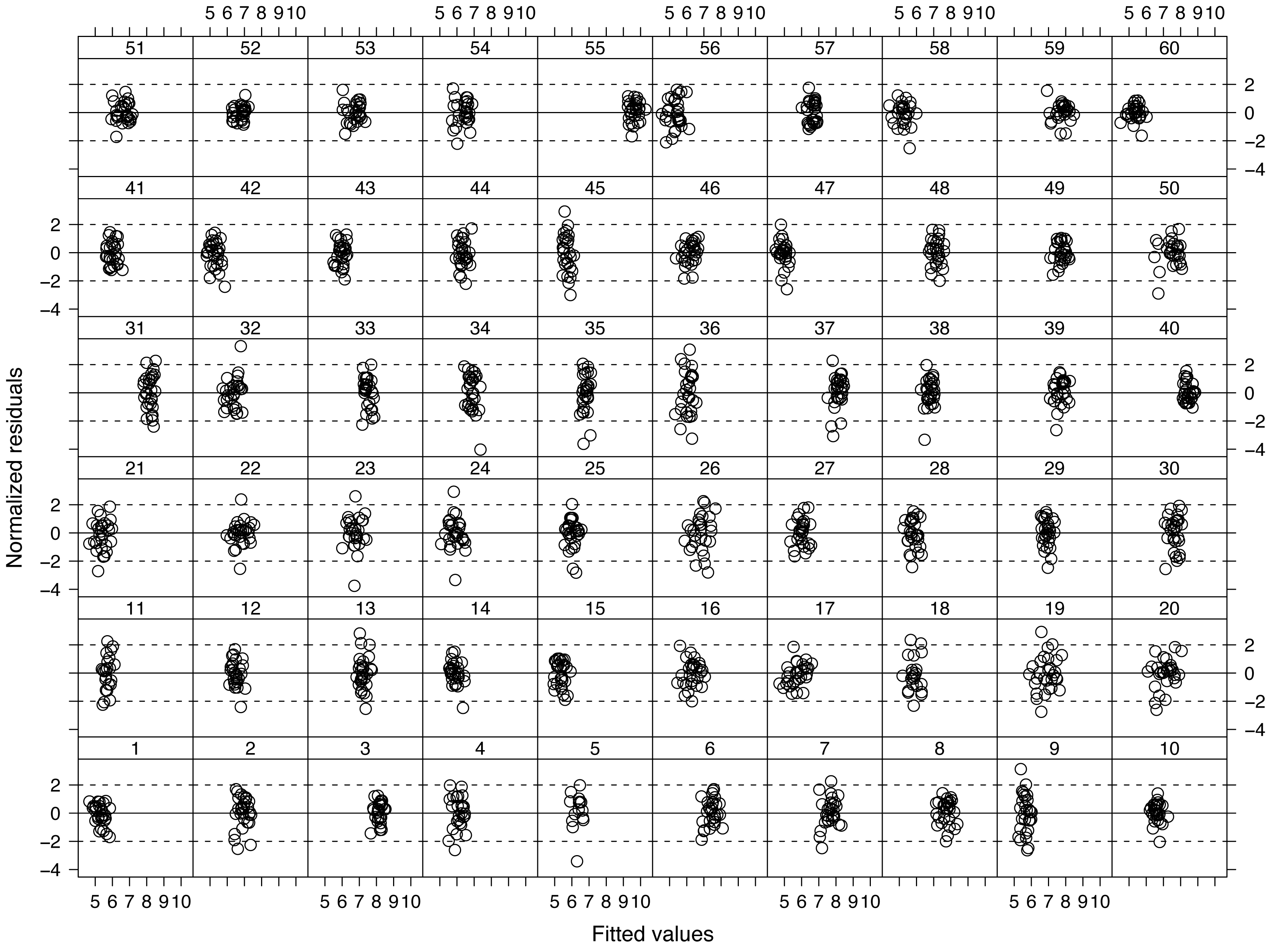

Supplement: Figure S3 — Normalized residuals versus fitted values by river. Numbers correspond to ‘ID’ column in Table S1. (TIF) [file pone.0024005.s003.tif]

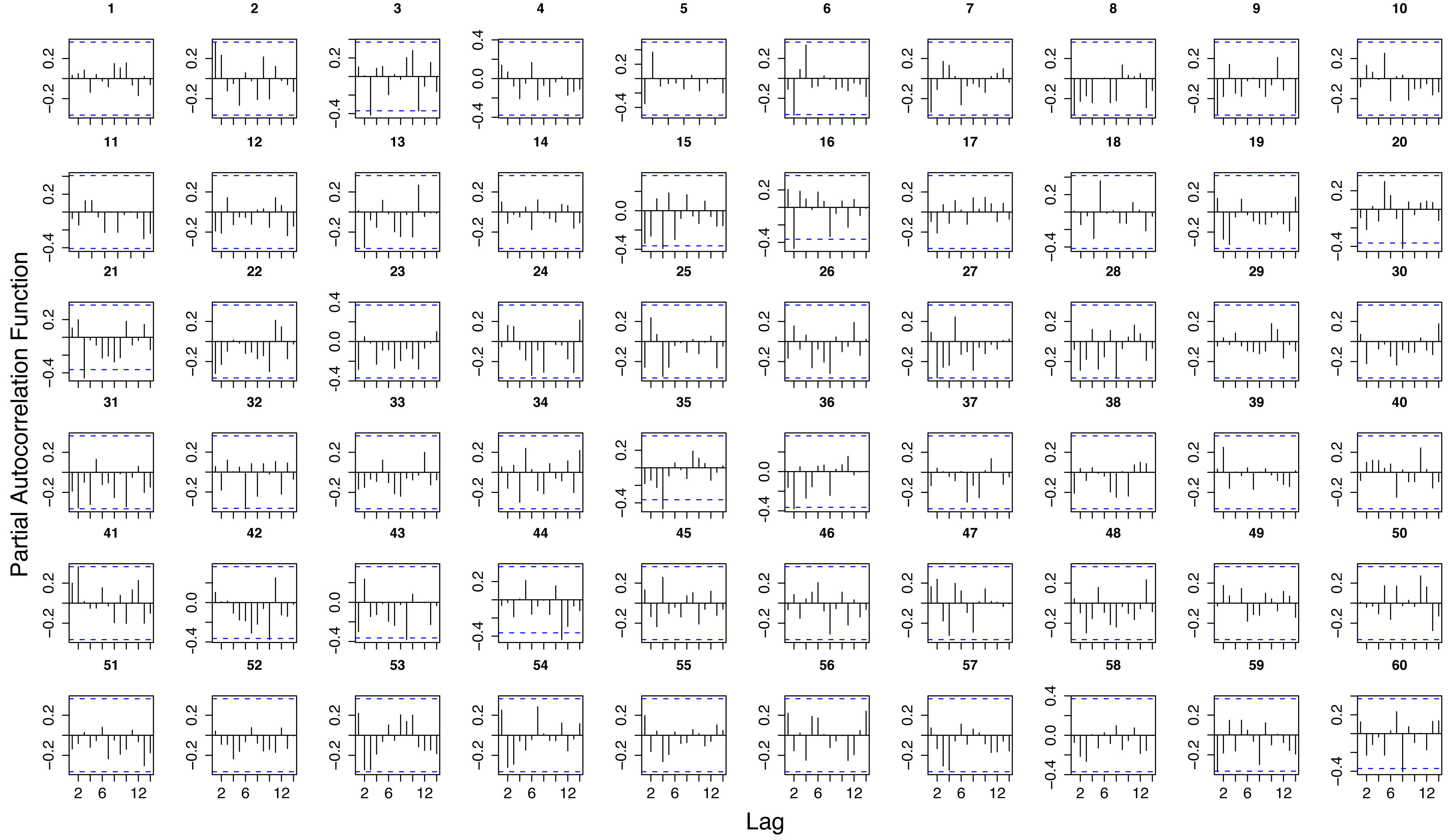

Supplement: Figure S4 — Partial Autocorrelation Function of the normalized residuals for each river. Numbers correspond to ‘ID’ column in Table S1. (TIF) [file pone.0024005.s004.tif]

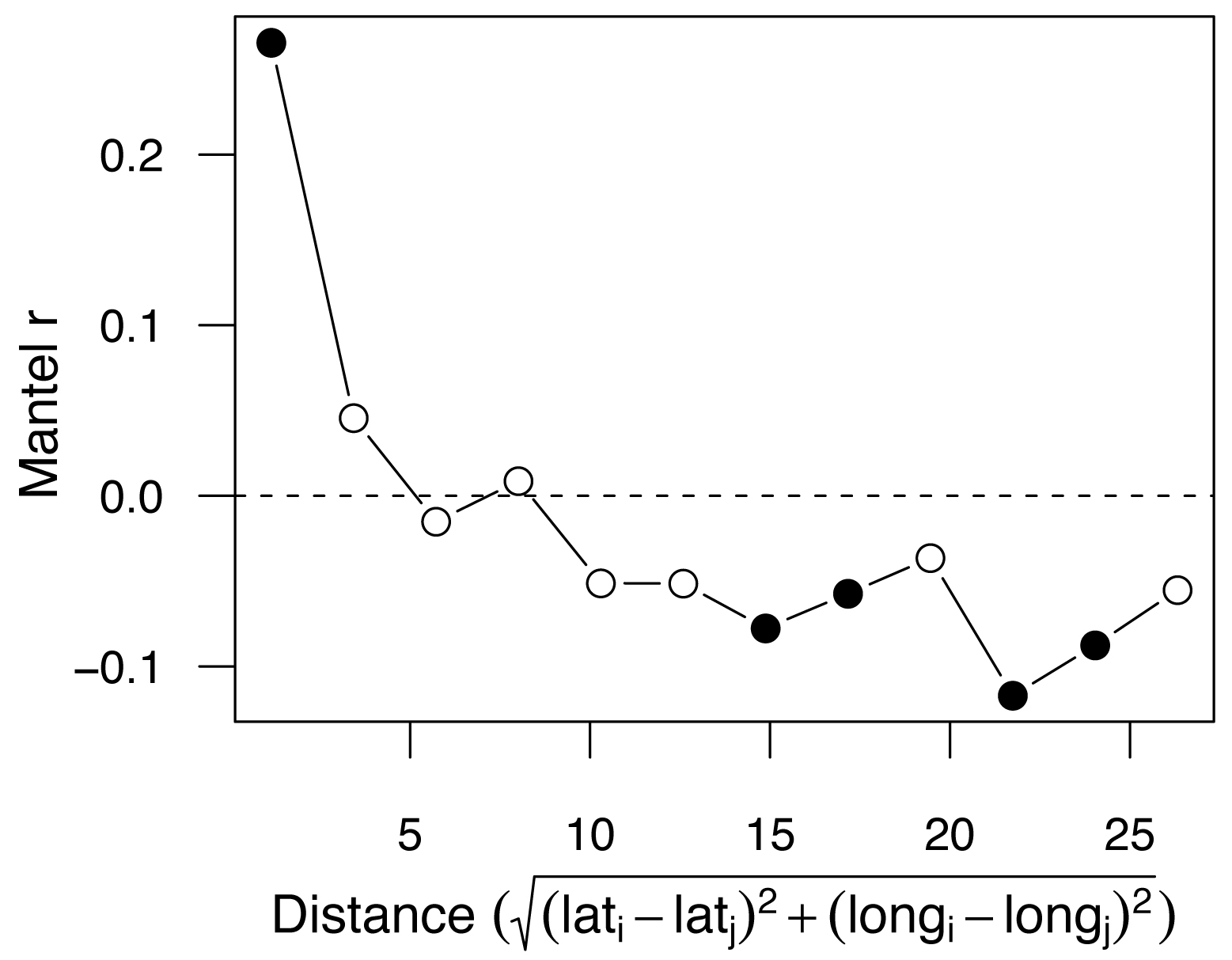

Supplement: Figure S5 — Spatial similarities. Mantel correlogram computed for 12 geographical distance classes on the 60 normalized residual time series. Positive values of the Mantel correlation indicate that similarity within that class of distance is higher than average, whereas, it is negative when the similarity is lower. Filled dots indicate significant results (P<0.05) of the Mantel test for a given distance class. (TIF) [file pone.0024005.s005.tif]

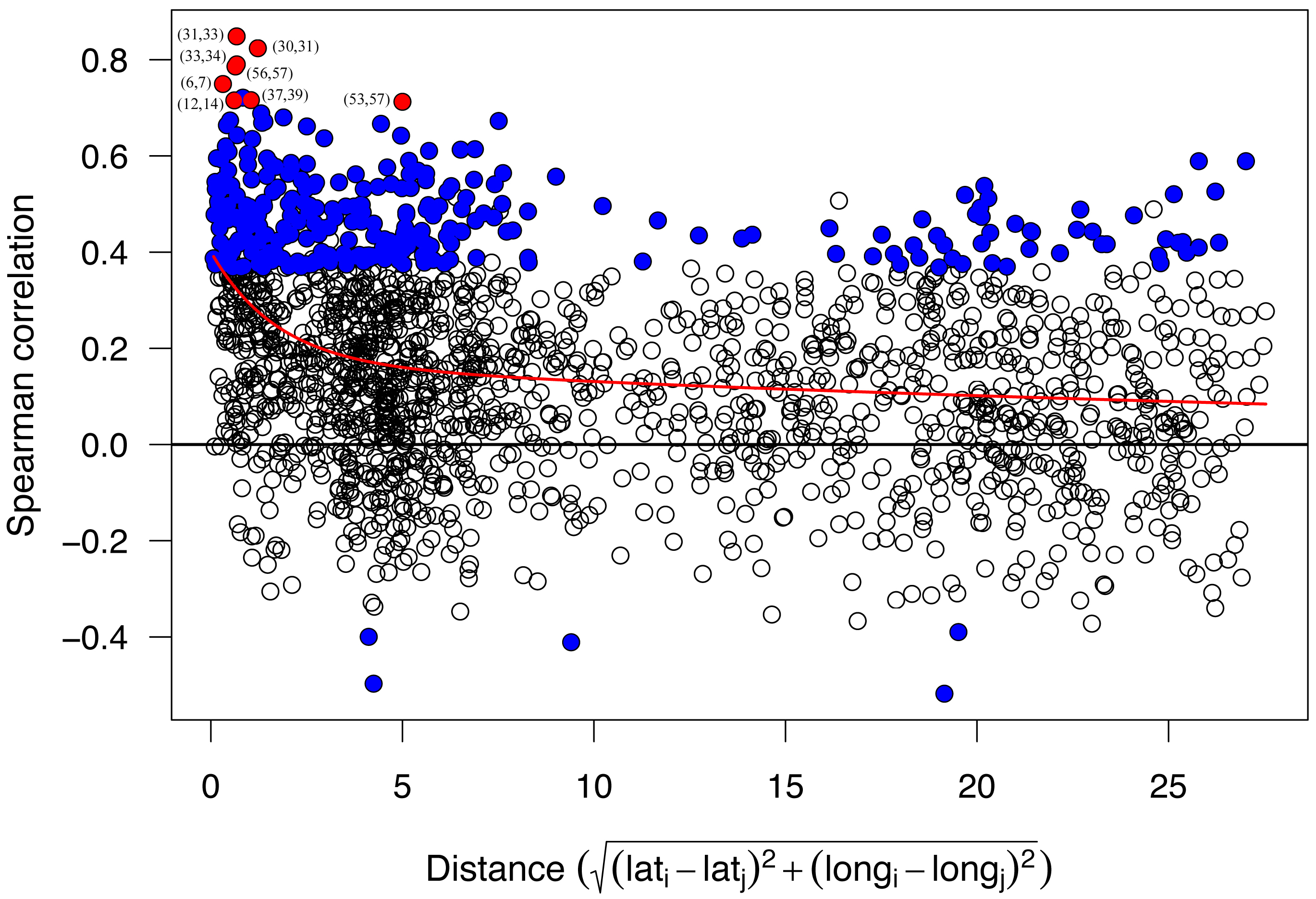

Supplement: Figure S6 — Pairwise Spearman correlations between river's normalized residuals and geographical distance (i.e. 1770 possible combinations). Open black circles indicate non-significant correlations; blue dots show significant correlations (301), and red dots show the significant pair correlations after Bonferroni correction (8; the corresponding rivers' number is indicated in brackets). The red line shows the fitted curve from a nonlinear model using generalized least squares (gnls). The average Spearman correlation was 0.16 ranging from –0.52 to 0.85 with 50% of the values between 0.02 and 0.31. Note also, that fitting variograms to the normalized residuals of the optimal model per year did not show any spatial correlation. (TIF) [file pone.0024005.s006.tif]

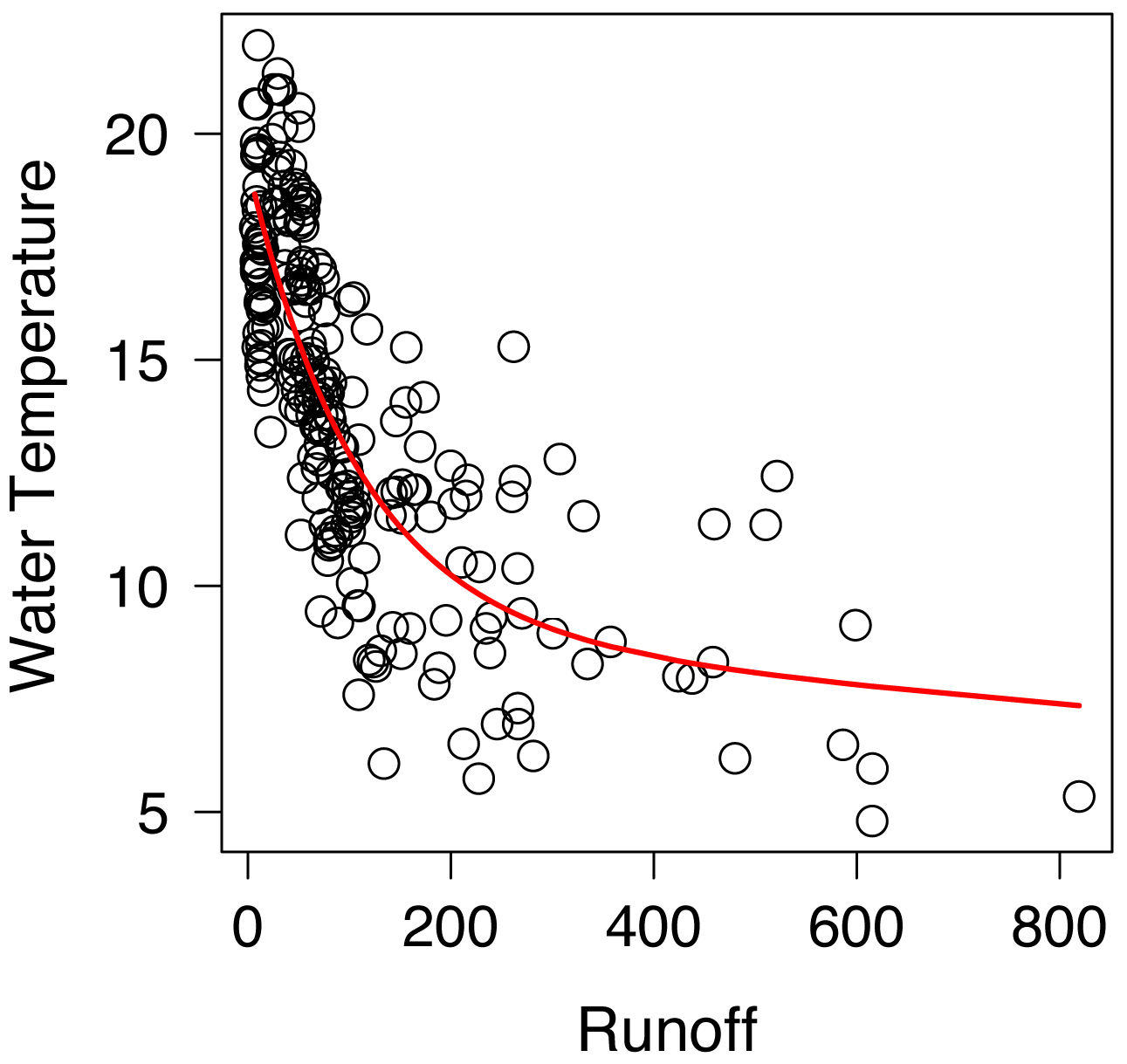

Supplement: Figure S7 — Water temperature and runoff. Plot of water temperature (°C) against runoff (m3 s–1) during upstream migration months (June–August) measured in six Norwegian rivers (Tovdalselva, Audna, Vosso, Gaula, Jølstra and Vefsna) from 1992 to 2007. The fitted curve from a biexponential model is also shown. (TIF) [file pone.0024005.s007.tif]

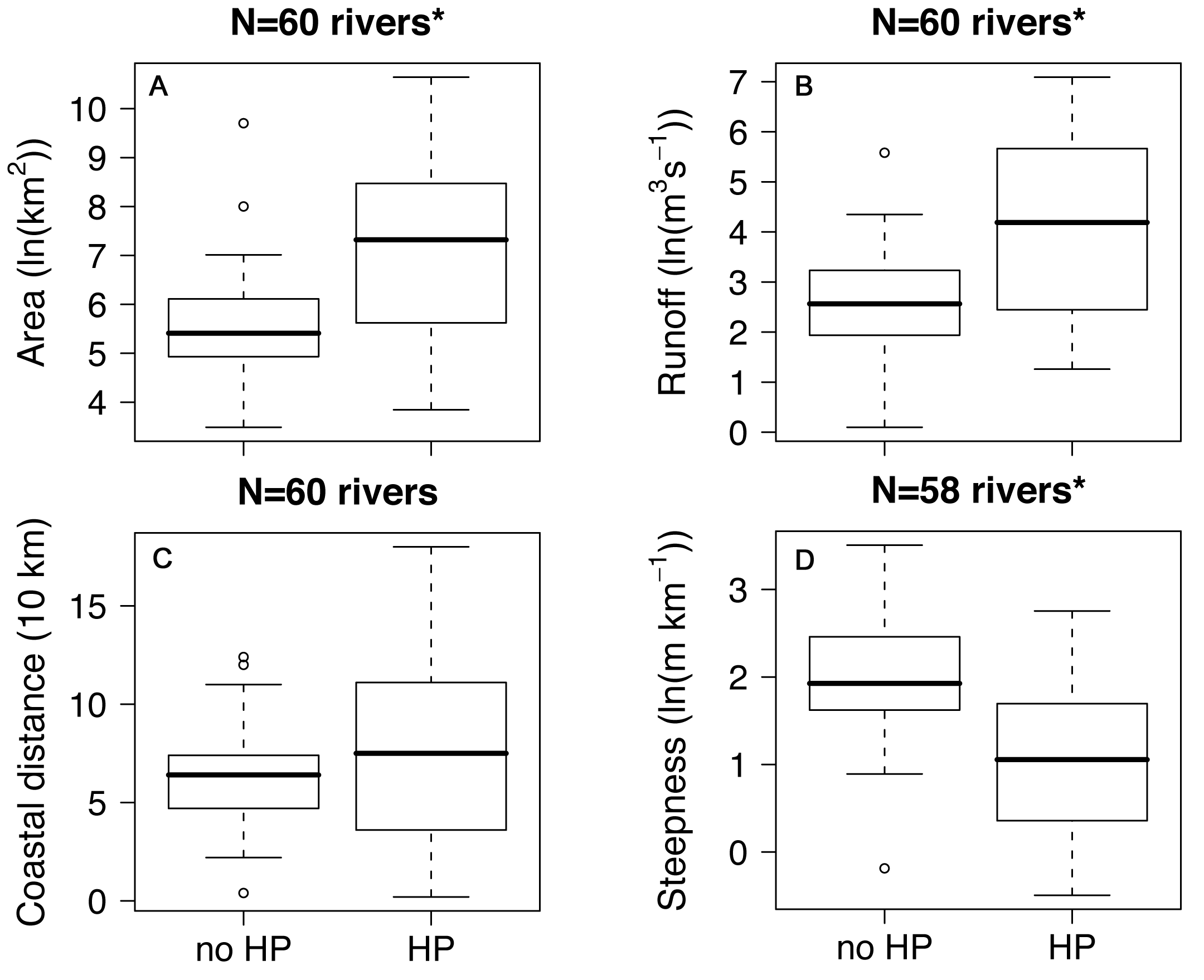

Supplement: Figure S8 — Rivers' characteristics. Box plots showing the variation in: (A) natural log-transformed catchment areas, (B) natural log-transformed mean water flow during upstream migration, (C) coastal migration distance (distance from river mouth to the coastal shelf), and (D) natural log-transformed steepness (ratio between altitude and river length) for the Norwegian rivers analyzed in this study with no hydropower stations (no HP) and the presence of at least one hydroelectric scheme along the salmon-producing part of the river (HP). The number of rivers with available data used in each plot appears on top of each graph. The asterisks indicate that differences are statistically significant according to an analysis of variance. (TIF) [file pone.0024005.s008.tif]
