# Supplementary material for: Quantifying the Ocean, Freshwater and Human Effects on Year-to-Year Variability of One-Sea-Winter Atlantic Salmon Angled in Multiple Norwegian Rivers
Source: PLoS One. 2011 Aug 29;6(8):e24005. doi: 10.1371/journal.pone.0024005 (PMC3163678; doi:10.1371/journal.pone.0024005)
Supplement: Table S1 — List of the rivers which interannual grilse catches were analyzed in this study. Latitude (N) and longitude (E) give the geographical position of each river mouth. Overall mean ±SD (kg), and number of observations (years), with the number of missing years between parentheses, are also shown. The last column indicates the first year (yy) in the time series with hydropower development (HP) and/or salmon farming (F). ‘no’ denotes non-presence of that activity for a given river during the studied period. (PDF) [file pone.0024005.s009.pdf]

Table S1. List of the rivers which interannual grilse catches were analyzed in this study. Latitude (N) and longitude (E) give the geographical position of each river mouth. Overall mean ±SD (kg), and number of observations (years), with the number of missing years between parentheses, are also shown. The last column indicates the first year (yy) in the time series with hydropower development (HP) and/or salmon farming (F). 'no' denotes non-presence of that activity for a given river during the studied period.

ID	River	Latitude	Longitude	Mean catch	SD	N	HP / F
1	Glomma	59º12'	10º57'	203.32	77.95	29 (4)	(79, no)
2	Drammenselva[b]	59º44'	10º14'	1697.04	974.21	29 (1)	(79, no)
3	Numedalslågen[d]	59º02'	10º03'	4389.86	2021.66	28 (0)	(79, no)
4	Skienselva[c]	59º07'	9º37'	593.7	422.27	27 (0)	(80, no)
5	Sokndalselva	58º19'	6º17'	937.27	613.91	15 (0)	(93, no)
6	Ogna[a]	58º31'	5º48'	2494.76	1569.77	29 (0)	(79, no)
7	Håelva	58º41'	5º32'	3299.34	2014.47	29 (0)	(79, no)
8	Figgjo	58º48'	5º33'	3200.76	1627.27	29 (0)	(79, no)
9	Suldalslågen[a, d]	59º29'	6º15'	357.28	246.71	29 (0)	(79, 79)
10	Etneelva	59º40'	5º56'	955.24	534.46	29 (0)	(79, 79)
11	Eikefetelva	60º43'	5º33'	425.48	420.51	23 (0)	(no, 81)
12	Gaular	61º22'	5º41'	764.62	344.43	29 (0)	(no, no)
13	Nausta	61º31'	5º44'	1986.66	1819.62	29 (0)	(no, no)
14	Eidselva	61º54'	5º59'	446.17	220.83	29 (0)	(79, no)
15	Ervikelva	62º10'	5º07'	241	134.2	29 (0)	(no, 79)
16	Åheimselva[d]	62º03'	5º37'	702.25	471.17	29 (1)	(79, 79)
17	Austefjordelva	62º04'	6º19'	397.79	325.72	29 (0)	(79, 79)
18	Storelva	62º08'	6º15'	419.77	484.22	22 (0)	(no, 79)

19	Ørstaelva	62º11’	6º08’	1510.5	1391.87	29 (1)	(no, 79)
20	Bondalselva	62º12’	6º28’	2075.14	2254.72	29 (1)	(no, 79)
21	Vikelva	62º06’	6º34’	267.11	209.44	29 (1)	(79, 79)
22	Velledalselva	62º20’	6º36’	1161.52	745.09	29 (0)	(no, 79)
23	Strandaelva	62º18’	6º56’	1265.76	1276.21	28 (3)	(no, 79)
24	Korsbrekkelva[b]	62º05’	6º52’	479.32	444.5	29 (1)	(no, 79)
25	Stordalselva	62º27’	6º59’	507.45	313.27	29 (0)	(no, no)
26	Oselva	62º48’	7º43’	1704.97	1967.96	29 (0)	(no, 79)
27	Sylteelva	62º50’	7º12’	564.33	572.23	29 (2)	(no, 79)
28	Søya	62º53’	8º32’	390.43	260.01	29 (1)	(no, no)
29	Surna	62º58’	8º40’	1396.9	933.38	29 (0)	(79, no)
30	Orkla[d]	63º19’	9º50’	3202.24	2530.16	29 (0)	(82, no)
31	Gaula	63º21’	10º14’	5234.79	3424.58	29 (0)	(79, no)
32	Nidelva	63º26’	10º24’	877.03	876.71	29 (0)	(79, no)
33	Stjørdalselva	63º26’	10º54’	2388.24	1163	29 (0)	(79, no)
34	Verdalsvassdraget	63º48’	11º28’	1499.69	1003.42	29 (0)	(90, no)
35	Skauga	63º36’	9º56’	1248.52	993.73	29 (0)	(79, no)
36	Nordelva	63º46’	10º07’	669.08	621.94	29 (4)	(79, no)
37	Stordalselva	63º57’	10º13’	5319.48	3723.88	29 (0)	(no, 79)
38	Steinselva	64º18’	10º31’	1192.75	812.92	29 (1)	(no, 79)
39	Årgårdselva	64º18’	11º12’	3553.34	2492.31	29 (0)	(no, no)
40	Namsen	64º28’	11º35’	5928.72	2863.46	29 (0)	(79, 79)
41	Salvassdraget	64º42’	11º26’	512.72	343.67	29 (0)	(no, 79)
42	Kongsmoelva	64º53’	12º27’	221.86	141.88	29 (0)	(no, no)
43	Åelva	65º05’	12º27’	410.32	237.01	29 (4)	(79, 83)

44	Vefsna[b]	65º50’	13º13’	664.59	551.1	29 (0)	(79, 79)
45	Ranavassdraget[b]	66º20’	14º09’	386.46	280.45	29 (3)	(79, 79)
46	Laukhellevassdraget	69º14’	17º51’	606.97	376.13	29 (0)	(no, 83)
47	Lakselva (Aursfjorden)	69º17	18º43’	186.12	137.14	29 (3)	(79, 79)
48	Målselva	69º14’	18º31’	1749.9	1018.45	29 (0)	(79, no)
49	Altaelva	69º58’	23º23’	2798.34	1528.16	29 (0)	(87, 83)
50	Repparfjordelva	70º27’	24º20’	2257.93	1368.6	29 (0)	(no, 84)
51	Stabburselva	70º11’	24º54’	915.62	522.32	29 (0)	(no, no)
52	Lakselva	70º04’	24º55’	990.75	545.77	29 (1)	(79, no)
53	Børselva	70º18’	25º32’	979.9	508.01	29 (0)	(no, no)
54	Langfjordelva	70º37’	27º36’	731	438.22	29 (2)	(no, no)
55	Tanaelva	70º28’	28º20’	21 437.38	10 443.22	29 (0)	(no, no)
56	Vesterelva	70º32’	29º58’	294.66	401	29 (0)	(no, no)
57	Komagelva	70º14’	30º32’	876.52	444.58	29 (0)	(no, no)
58	Munkelva	69º39’	29º27’	192.5	106.88	28 (0)	(no, 83)
59	Neidenelva	69º42’	29º24’	3033.83	1282.49	29 (0)	(no, 83)
60	Karpelva	69º40’	30º23’	251.81	129.75	28 (1)	(no, 83)

[a]Rivers with liming within the studied years.
[b]Rivers infected with *Gyrodactylus salaris* within any of the studied years.
[c]Stocking programmes started within the study period.
[d]Presence of fish ladders built within the study period.
