# Supplementary material for: Quantifying the Ocean, Freshwater and Human Effects on Year-to-Year Variability of One-Sea-Winter Atlantic Salmon Angled in Multiple Norwegian Rivers
Source: PLoS One. 2011 Aug 29;6(8):e24005. doi: 10.1371/journal.pone.0024005 (PMC3163678; doi:10.1371/journal.pone.0024005)
Supplement: Table S2 — Median dates as well as earliest and latest dates of smolt run of Atlantic salmon in some Norwegian watersheds over several years. The descent date is defined as the day each year when 50% of the smolts have passed the gauging station. (PDF) [file pone.0024005.s010.pdf]

Table S2. Median dates as well as earliest and latest dates of smolt run of Atlantic salmon in some Norwegian watersheds over several years. The descent date is defined as the day each year when 50% of the smolts have passed the gauging station.

ID	River	Location	Smolt run (annual range)	Years	Period	Reference
3	Numedalslågen	59º02'N 10º03'E	4 May (29 April–10 May)	3	2003–2005	Forseth, T. (pers. comm.)
ni	Tovdalselva	58º12'N 8º04'E	17 May (3 May–23 May)	4	2004–2007	Hesthagen (2008)
ni	Mandalselva	58º01'N 7º27'E	8 May (30 April–21 May)	4	2004–2007	Hesthagen (2008)
ni	Imsa	58º54'N 5º58'E	15 May (7 May–25 May)	20	1976–1995	Hvidsten *et al*. (1998)
9	Suldalslågen	59º29'N 6º15'E	1 May (27 April–5 May)	10	1995–2004	Saltveit (2004)
ni	Guddalselva	59º58'N 6º00'E	2 May (28 April–17 May)	4	2005–2008	Skaala, Ø. (pers. comm.)
ni	Vosso	60º38'N 5º57'E	15 May (11 May–26 May)	7	2001–2007	Barlaup (2008)
ni	Flåm	60º52'N 7º07'E	21 May (8 May–9 June)	5	2002–2006	Sægrov *et al*. (2007)
ni	Aurland	60º54'N 7º12'E	26 May (8 May–6 June)	5*	2001–2006	Sægrov *et al*. (2007)
ni	Eira	62º41'N 8º08'E	13 May (6 May–24 May)	8	2001–2008	Jensen *et al*. (2009)
30	Orkla	63º19'N 9º50'E	15 May (6 May–4 June)	22	1983–2006	Hvidsten *et al*. (2009)
33	Stjørdalselva	63º26'N 10º54'E	23 May (12 May–7 June)	15	1991–2006	Arnekleiv *et al*. (2007)

ni	Saltdal	67º06’N 15º25’E	5 June (23 May−19 June)	6	1990−1995	Hvidsten *et al*. (1998)
ni	Hals	70º02’N 22º58’E	21 June (11 June−5 July)	16	1988−2004	Hvidsten *et al*. (2009)
49	Altaelva	69º58’N 23º23’E	25 June (17 June−11 July)	7	1989−1995	Hvidsten *et al*. (1998)
55	Tanaelva	70º28’N 28º20’E	Early July (median June−early July)	6	2002−2007	Niemelä *et al*. (2009)

ni: indicates that the river is not included in the current analyses of grilse catches.
*No data was gathered in 2004.

References

Arnekleiv JV, Rønning L, Koksvik J, Kjærstad G, Alfredsen K, et al. (2007) Ferskvannsbiologiske undersøkelser i Stjørdalselva 1990-2006. Faglig oppsummering: kraftverksregulering, bunndyr, drivfauna, ungfisk og smolt. NTNU Vitenskapsmuseet Rapport Zoologisk Serie 2007 No. 1: 141 p. (In Norwegian). Available: http://www.ntnu.no/c/document_library/get_file?uuid=abd35162-1488-4050-afe3-25f6a12b8f20&groupId=10476

Barlaup B (2008) Nå eller aldri for vossolaksen – anbefalte tiltak med bakgrunn i bestandsutvikling og trusselfaktorer. Direktoratet for Naturforvaltning Utredning 2008 No. 9: 176 p. (In Norwegian). Available: http://www.nina.no/archive/nina/PppBasePdf/Kapitler/2008/Schartau%20Forsuring%20DNUtredning%209%202008%20kap%207.pdf

Hesthagen T (2008) Reetablering av laks på Sørlandet. Årsrapport fra reetableringsprosjektet 2007. Direktoratet for Naturforvaltning Utredning 2008 No. 8: 63 p. (In Norwegian). Available: http://www.dirnat.no/content.ap?thisId=631

Hvidsten NA, Heggberget TG, Jensen AJ (1998) Sea water temperatures at Atlantic salmon smolt enterance. Nordic J Fresh Res 74: 79–86.

Hvidsten NA, Jensen AJ, Rikardsen AH, Finstad B, Aure J, et al. (2009) Influence of sea temperature and initial marine feeding on survival of Atlantic salmon *Salmo salar* post-smolts from the Rivers Orkla and Hals, Norway. J Fish Biol 74: 1532−1548.

Jensen AJ, Bremset G, Finstad B, Hvidsten NA, Jensås JG, et al. (2009) Fiskebiologiske undersøkelser i Auravassdraget. Årsrapport 2008. NINA Rapport No. 451: 53 p. (In Norwegian). Available: http://www.nina.no/archive/nina/PppBasePdf/rapport/2009/451.pdf

Niemelä E, Hassinen E, Muladal R, Brørs S, Sandring S (2009) Den atlantiske laksen (*Salmo salar*, L.) i Tanavassdraget I: miljøforholdene i den subarktiske Tanavassdraget og virkningen av dem på laksefisket og laksen. Fylkesmannen i Finnmark Rapport No. 5: 45 p. (In Norwegian). Available: http://www.fylkesmannen.no/fagom.aspx?m=1887&amid=1001566

Sægrov H, Hellen BA, Kålås S, Urdal K, Johnsen GH (2007) Endra manøvrering i Aurland 2003-2006. Sluttrapport – Fisk. Rådgivende Biologer Rapport No. 1000: 103 p. (In Norwegian). Available: http://www.e-co.no/filestore/Aurlandselvafiskeunderskelser2000-2007RdgBSluttrapport.pdf

Saltveit SJ (2004) Smoltutvandring hos laks i Suldalslågen 2004. Rapp. Lab. Ferskv. Økol. Innlandsfiske, Oslo No. 235: 30 p. (In Norwegian). Available: http://www.statkraft.no/Images/Smoltutvandring%20hos%20laks%20i%20Suldalslågen%202004_tcm10-4164.pdf
