# Supplementary material for: Quantifying the Ocean, Freshwater and Human Effects on Year-to-Year Variability of One-Sea-Winter Atlantic Salmon Angled in Multiple Norwegian Rivers
Source: PLoS One. 2011 Aug 29;6(8):e24005. doi: 10.1371/journal.pone.0024005 (PMC3163678; doi:10.1371/journal.pone.0024005)
Supplement: Table S3 — Summary of the variables used in this large-scale analysis ( n = 60 one-sea-winter Atlantic salmon time series). (PDF) [file pone.0024005.s011.pdf]

Table S3. Summary of the variables used in this large-scale analysis (*n* = 60 one-sea-winter Atlantic salmon time series).

Variable	Mean	Range	Brief description
Catch (C, in kg)	1776	25–45 020	Total catch (mainly rod) of one-sea-winter fish (1SW).
Temperature (SST, in ºC)	8.09	5.83–13.00	Coastal sea surface temperature expected to influence smolts entering the ocean.
Runoff (R, in m^3 s^{-1})	99.16	0.69–1618	Water flow expected to affect upstream migration.
Coastal fishery (SF, in %)	56.59	2.09–98.31	Proportion of 1SW caught in the coastal ocean relative to total 1SW catches (sea fishery and recreational fishery in rivers).
Time (Y, in yr)	1993	1979–2007	Time trend of catches.
Farming (F)	29*	na	Presence/absence of net pens potentially influencing smolts on their way to the open ocean.
Damming (HP)	28**	na	Presence/absence of hydroelectric stations potentially influencing upstream migration.

na: not applicable.
*Number of rivers out of 60 draining in areas with at least the presence of one farming license established during any year of the study period.
**Number of rivers out of 60 that contained at least one hydroelectric scheme along the salmon-producing part of the river.
