# Supplementary material for: Quantifying the Ocean, Freshwater and Human Effects on Year-to-Year Variability of One-Sea-Winter Atlantic Salmon Angled in Multiple Norwegian Rivers
Source: PLoS One. 2011 Aug 29;6(8):e24005. doi: 10.1371/journal.pone.0024005 (PMC3163678; doi:10.1371/journal.pone.0024005)
Supplement: Table S4 — Comparison of different models showing the number of parameters, Bayesian Information Criteria (BIC) and the difference in BIC values between each model and the model with the optimal random structure. First, we selected the appropriate random effects (Models 1 to 5) where the fixed component contained all explanatory variables and reasonable interactions. Then, different correlation structures (Autoregressive: AR, Moving Average: MA) for modeling within-group serial correlation were compared (Models 6 to 8), and heteroscedasticity was handled by modeling the residual variance as an exponential function of runoff (Model 9). (PDF) [file pone.0024005.s012.pdf]

Table S4. Comparison of different models showing the number of parameters, Bayesian Information Criteria (BIC) and the difference in BIC values between each model and the model with the optimal random structure. First, we selected the appropriate random effects (Models 1 to 5) where the fixed component contained all explanatory variables and reasonable interactions. Then, different correlation structures (Autoregressive: AR, Moving Average: MA) for modeling within-group serial correlation were compared (Models 6 to 8), and heteroscedasticity was handled by modeling the residual variance as an exponential function of runoff (Model 9).

Model nº	Random effects	Correlation structure	Parameters	BIC	ΔBIC
1	none	none	12	5274.94	1628.64
2	β_0	none	13	4036.39	390.01
3	β_0 and β_3	none	14	4041.58	395.28
4	β_0 and β_6	none	14	3889.74	243.44
5	β_0, β_3 and β_6	none	15	3895.69	249.40
6	β_0 and β_6	AR(1)	15	3662.06	15.76
7	β_0 and β_6	MA(1)	15	3718.94	72.64
8	β_0 and β_6	ARMA(1,1)	16	3649.36	3.06
9	β_0 and β_6	ARMA(1,1)	17	3646.30	0

Note that, once the optimal random structure has been found (i.e. Model 9) using restricted maximum likelihood estimation (REML), the optimal fixed components were selected using maximum likelihood estimation (ML). The parameters of the final model presented in Table 1 in the main text were finally obtained using REML.
