# Supplementary material for: Quantifying the Ocean, Freshwater and Human Effects on Year-to-Year Variability of One-Sea-Winter Atlantic Salmon Angled in Multiple Norwegian Rivers
Source: PLoS One. 2011 Aug 29;6(8):e24005. doi: 10.1371/journal.pone.0024005 (PMC3163678; doi:10.1371/journal.pone.0024005)
Supplement: Discussion S1 — Further details on catch data and model robustness. (PDF) [file pone.0024005.s013.pdf]

Discussion S1. Further details on catch data and model robustness.

Catch data

The quality of the fishery database can be further discussed. We recognize various concerns regarding our set of data. First, there is probably some underreporting of catch and this might also vary among years and watercourses. In a study from two Norwegian rivers it was shown that those that did not report the catches fished approximately one third of those that reported their catch (Fiske and Aas, 2001). Second, there are obvious differences in capture efficiency between fishers. According to studies in four Norwegian rivers, local fishers capture on average more salmon each day using less effort (fishing hours) than non-locals (Fiske and Aas, 2001). In general, it can be assumed that local fishers are more prone to report their catch than non-locals. Therefore, we assumed that reported fish are a random sample of the fish actually captured and that fishers report fish independently of size. Third, we are aware that recent studies show that the behavior of fishermen is decisive for the dynamics of recreational freshwater fisheries (e.g. Post *et al.*, 2008). However, no quantitative data are available on the behavior of salmon anglers in Norway. Fourth, we also assumed that the reported catch data would reflect the population variability and that it contains useful information about changes in salmon abundance (e.g. Friedland *et al.*, 2003; Thorley *et al.*, 2005). For ten Norwegian rivers it has been shown that fishers catch between 30–60% of the fish that are available for capturing, and that this proportion tends to be rather stable over time (1985–2000; these last data are from three rivers only) (Fiske and Aas, 2001; Hansen, 1990). On the other hand, the proportion of salmon captured is for some Norwegian rivers negatively correlated with the number of ascending salmon (Fiske and Aas, 2001). The same has been reported for Pacific salmon (Peterman and Steer, 1981), whereas Crozier and Kennedy (2001) report no

such correlation for river Bush in Northern Ireland. Such a negative correlation may lead to overestimations when abundance is low and underestimations when abundance is high. Overall this may influence our ability to detect trends in the data. Fifth, Niemelä (2004) reported for the river Tanaelva that catch per unit of effort has remained stable during the period 1974–2003 indicating no changes in fishing efficiency within the largest river in the area. Finally, our modeling approach does not take into account factors affecting life stages previous to smolting, thus, the results of those effects would be embedded in the catch time series. Nevertheless, it is worth noting that the strength of the smolt year class would be related to subsequent sea-age groups (e.g. Niemelä *et al.*, 2005).

Model robustness

Exclusions of data

It is well documented that several other human encroachments not taken into account in our analysis can affect salmonid populations. For instance, acidification of freshwater in the southern part of Norway led to strong reductions, and even extinctions, of several Atlantic salmon populations (Hesthagen and Hansen, 1991). Several of these rivers have been restored through various mitigating management schemes (mainly through liming efforts, e.g. Hesthagen and Larsen, 2003). Moreover, parasitic infestations as those induced by the monogenean *Gyrodactylus salaris* led to rapid and dramatic decline in some Atlantic salmon populations (e.g. Johnsen and Jensen, 1986). On the other hand, stock enhancement (stocking, fish ladder constructions) could have positive effects on the populations (e.g. L'Abée-Lund *et al.*, 2006). During the study period, 10 of the rivers investigated here were influenced by one of the above factors (see Table S1); however, we considered the effects likely to be minor at the large scale of the study. Indeed, excluding the affected rivers to test for any effect they might have

had on our results did not change our conclusions. Moreover, excluding three rivers where fixed and seine gears were allowed during the study period did not vary our results either. Furthermore, for 15 rivers, the data indicated that the reporting of the various weight-groups was biased in the years 1979–1982 due to a change in reporting procedure that probably was not effectively implemented in all Norwegian rivers (L'Abée-Lund *et al.*, 2004). To reduce any effects of biased weight categorization, and to err on the conservative side, we re-ran our statistical model excluding the years 1979–1982 for those rivers for which the data was in doubt. Our results were robust to this exclusion. Finally, conclusions did not change either by setting a minimum catch of 10 individuals per year that resulted in 85 rivers to be analyzed (see subsection 'Catch data' in Materials and Methods in the main text). This suggests that our results do not depend on the number of fish reported.

Time series interpolation

Some time series (18) had missing values (35 out of 1707) (see Table S1 and Figure S1); therefore they were interpolated using a procedure based on a singular spectrum analysis (Ibañez and Etienne, 1992). Nevertheless, running the models excluding the interpolated series, or not filling the gaps, did not alter the results.

Farming licenses as a continuous covariate

To determine the potential impact of salmon farming in net pens on smolts on their way to the open ocean we used presence/absence of registered licenses in each Norwegian municipality. This approach was used to err on the conservative side due to some uncertainties in this data set, that is, a given license usually owns multiple net pens but the net pens might not all work simultaneously (i.e. contain fish), in addition, each license might be allowed to operate in different municipalities. Ideally, the data should reflect the total annual production of farmed salmon –and/or parasite

abundance– per net pen but, unfortunately, to our knowledge, these data are not available. Nevertheless, we ran the final model using the number of licenses registered in each year at the municipality level as a covariate and the result was the same. The negative time trend in grilse catches was stronger for higher numbers of registered aquaculture licenses.

Simulations of effort

The purpose of the simulations was to assess the robustness of the coefficients of the optimal model when including a new covariate, 'effort', based on an ARIMA(3,1,0) model fitted to Tanaelva fishing days (Figure S9). As shown in the main text, coefficients did not depart from those obtained without including effort, and only in 4.6% of the cases, out of 1000 model runs, 'fishing effort' was significant. Coefficients from those significant (positive and negative) cases did not show any major departures from the values obtained with the model that did not include any effort term either; and there was not any simulated effort from a particular river driving these results. However, note that including another covariate (i.e. fixed-effect) once an optimal mixed model has been selected does not satisfy the model selection procedure followed in Table S4. Nevertheless, to test that including effort as a fixed-effect once the model is presumed to be optimal does not lead to wrong conclusions, we ran the model various times starting with a fixed component that included effort and then followed the steps depicted in Table S4 to select the random structure. In all cases starting with effort in the fixed part did not change the model structure. Therefore, we assume that including effort at a later stage does not imply ending up with different models. Finally, simulated effort does not co-vary with the other explanatory variables.

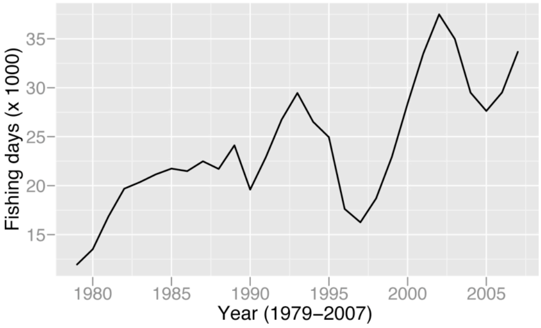

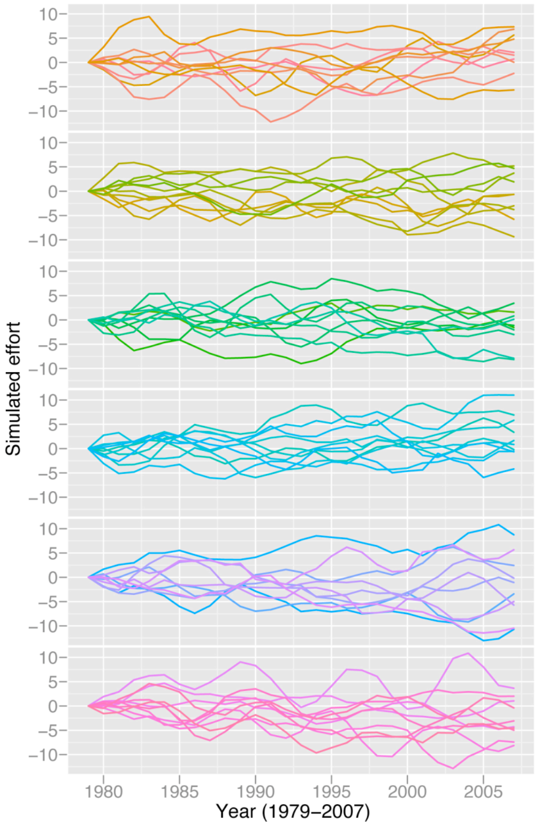

Figure S9. Time series of fishing days in the Finnish part of Tanaelva river (Erkinaro *et al.*, 2010); and multiple examples of simulated time series of effort based on an ARIMA(3,1,0) model fitted to Tanaelva fishing days.

References

Crozier WW, Kennedy GJA (2001) Relationship between freshwater angling catch of Atlantic salmon and stock size in the River Bush, Northern Ireland. J Fish Biol 58: 240–247.

Erkinaro J, Länsman M, Kuusela J, Niemelä E, Kylmäaho M, et al. (2010) Status of salmon stocks in the rivers Teno/Tana and Näätämöjoki/Neidenelva. ICES North

Atlantic Salmon Working Group. Working Paper 24. 10 p.

Fiske P, Aas Ø (2001) Laksefiskeboka. Om sammenhenger mellom beskatning, fiske og verdiskapning ved elvefiske etter laks, sjøaure og sjørøye. NINA Temahefte No. 20: 100 p. (In Norwegian). Available: http://www.nina.no/archive/nina/PppBasePdf/temahefte/2001/20.pdf

Friedland KD, Reddin DG, McMenemy JR, Drinkwater KF (2003) Multidecadal trends in North American Atlantic salmon (*Salmo salar*) stocks and climate trends relevant to juvenile survival. Can J Fish Aquat Sci 60: 563–583.

Hansen LP (1990) Exploitation of Atlantic salmon (*Salmo salar* L.) from the River Drammenselv, SE Norway. Fish Res 10: 125–135.

Hesthagen T, Hansen LP (1991) Estimates of the annual loss of Atlantic salmon, *Salmo salar* L., in Norway due to acidification. Aqua Fish Manag 22: 85–91.

Hesthagen T, Larsen BM (2003) Recovery and re-establishment of Atlantic salmon, *Salmo salar*, in limed Norwegian rivers. Fish Manag Ecol 10: 87–95.

Ibañez F, Etienne M (1992) Le filtrage des séries chronologiques par l'analyse en composantes principales de processus (ACPP). J Rech Océanogr 16: 27–33.

Johnsen BO, Jensen AJ (1986) Infestations of Atlantic salmon, *Salmo salar* L., by *Gyrodactylus salaris* in Norwegian rivers. J Fish Biol 29: 233–241.

L'Abée-Lund JH, Vøllestad LA, Beldring S (2004) Spatial and temporal variation in the grilse proportion of Atlantic salmon in Norwegian rivers. Trans Am Fish Soc 133: 743–761.

L'Abée-Lund JH, Haugen TO, Vøllestad LA (2006) Disentangling local from macroenvironmental effects: quantifying the effect of human encroachments based on historical river catches of anadromous salmonids. Can J Fish Aquat Sci 63: 2318–2329.

Niemelä E (2004) Variation in the yearly and seasonal abundance of juvenile Atlantic salmon in a long-term monitoring programme. Methodology, status of stock and reference points. PhD thesis, University of Oulu, Finland, 56 p. Available: http://herkules.oulu.fi/isbn9514273702/isbn9514273702.pdf

Niemelä E, Erkinaro J, Julkunen M, Hassinen E (2005) Is juvenile salmon abundance related to subsequent and preceding catches? Perspectives from a long-term monitoring programme. ICES J Mar Sci 62: 1617–1629.

Peterman RM, Steer GJ (1981) Relation between sport-fishing catchability coefficients and salmon abundance. Trans Am Fish Soc 110: 585–593.

Post JR, Persson L, Parkinson EA, van Kooten T (2008) Angler numerical response across landscapes and the collapse of freshwater fisheries. Ecol Appl 18: 1038–1049.

Thorley JL, Eatherley DMR, Stephen AB, Simpson I, MacLean JC, et al. (2005) Congruence between automatic fish counter data and rod catches of Atlantic salmon (*Salmo salar*) in Scottish rivers. ICES J Mar Sci 62: 809–817.
